# Identification and Functional Characterization of the Polymerizing Glycosyltransferase Required for the Transfer of d‑Ribose to the d‑Gal*f*NAc Moiety of the Capsular Polysaccharide of Campylobacter jejuni

**DOI:** 10.1021/acs.biochem.5c00052

**Published:** 2025-05-01

**Authors:** Dao Feng Xiang, Tamari Narindoshvili, Frank M. Raushel

**Affiliations:** Department of Chemistry, 14736 Texas A&M University, College Station, Texas 77843, United States

## Abstract

Campylobacter jejuni is the leading cause of food poisoning in the United States. The exterior surface of this bacterium is coated with a capsular polysaccharide (CPS) that helps protect the organism from the host immune system. In the HS:2 serotype of strain C. jejuni NCTC 11168, the minimal repeating trisaccharide consist of d-ribose, *N*-acetyl-d-galactosamine (GalNAc) and the serinol amide of d-glucuronic acid. Here we demonstrate that the C-terminal domain of Cj1432 (residues 574–914) is responsible for the transfer of d-ribose-5-P from phosphoribosyl pyrophosphate (PRPP) to C5 of the d-Gal*f*NAc moiety of the growing polysaccharide chain. In the next step the middle domain of Cj1432 (residues 357–573) catalyzes the hydrolysis of phosphate from this product. The N-terminal domain of Cj1432 (residues 1–356) catalyzes the transfer of d-GlcA from UDP-d-GlcA to C2 of the d-ribose moiety and thus Cj1432 catalyzes three consecutive reactions during the biosynthesis of the capsular polysaccharide of C. jejuni. We have previously shown that the remaining three reactions required for the polymerization of the CPS are catalyzed by the bifunctional enzyme Cj1438 and Cj1435. We have now demonstrated that the minimal repeating trisaccharide of the CPS of C. jejuni NCTC 11168 requires six enzyme-catalyzed reactions with six intermediate structures. This accomplishment will now enable the large-scale cell-free enzyme-catalyzed synthesis of well-defined oligomers of the CPS that can potentially be used in the production of glycoconjugate vaccines for the prevention of infections by C. jejuni.

## Graphical abstract

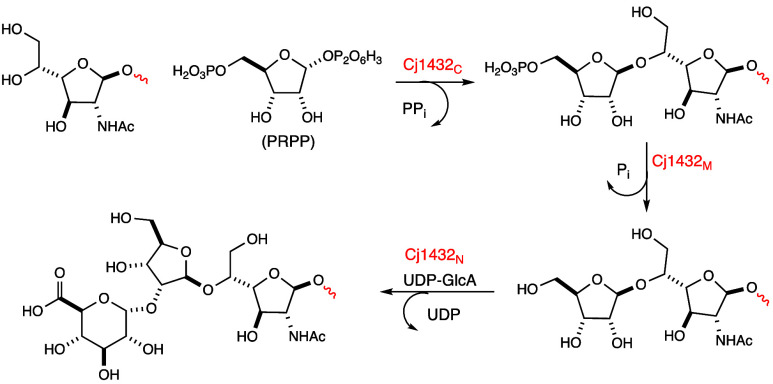

## Introduction

Campylobacter jejuni is the leading cause of food poisoning in the United States and Europe and is a major risk factor for the acquisition of the autoimmune disease, Guillan-Barré Syndrome.^1–3^ The primary route for infection is the consumption of raw or undercooked poultry or other contaminated foods. The CDC estimates that 1.5 million people in the United States get sick from *Campylobacter* infections each year, but many more cases go undiagnosed or unreported.^4–6^ Efforts to combat the bacterium with antibiotics have been hampered due to the development of multidrug resistance.^7–9^ Currently, there are no FDA-approved vaccines to prevent *Campylobacter* infections, and the best candidates are glycoconjugate vaccines, which enable the immune system to recognize surface-exposed sugars on the bacterium.^10^

The exterior surface of C. jejuni is coated with a capsular polysaccharide (CPS). The CPS helps to shield the bacterium from the host immune system and is also important for structural stability and maintenance of the cell wall.^11,12^ The CPS of C. jejuni is composed of repeating units of two to five monosaccharides that are attached to a poly Kdo (3-deoxy-d-*manno*-octulosonic acid) linker, which in turn is anchored to the outer cell wall through a covalent bond to diacylglycerol phosphate.^13,14^ The carbohydrate chain is further decorated by methylation, methyl phosphoramidylation, and/or amidation.^15,16^ At least 12 unique chemically determined CPS structures from more than 33 different C. jejuni serotypes have been identified thus far.^15,17^ The structure of the repeating CPS unit of C. jejuni NCTC 11168 (serotype HS:2) is shown in Figure 1 and the gene cluster for the biosynthesis of the CPS is presented in Figure 2.^18,19^

**Figure 1:**
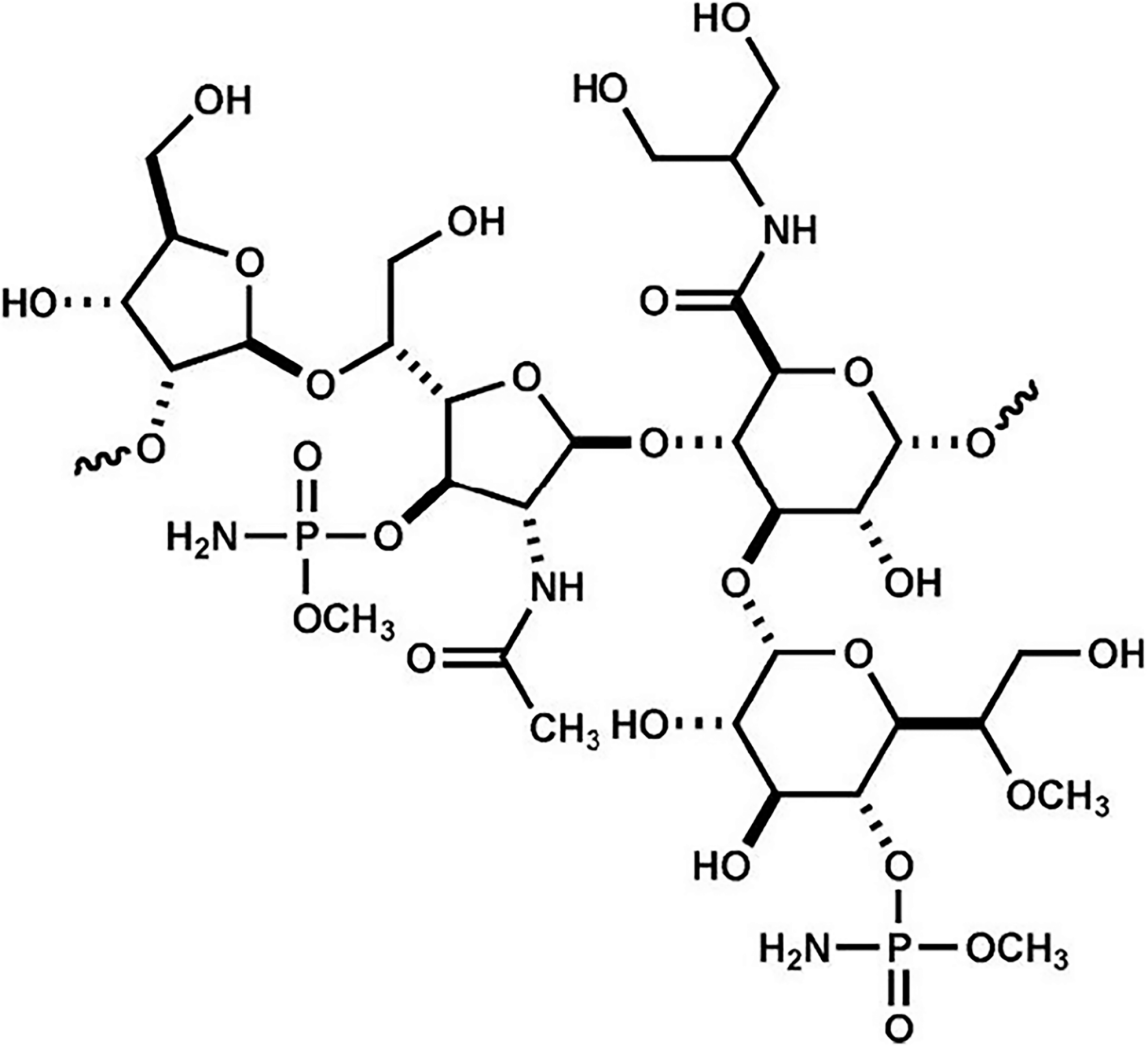
Structure of the repeating polysaccharide identified in the CPS of C. jejuni NCTC 11138 (serotype HS:2).

**Figure 2:**
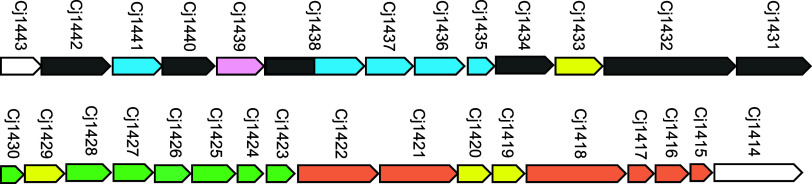
Gene cluster for the biosynthesis of the CPS in C. jejuni NCTC 11168 (serotype HS:2). The salmon-colored genes are responsible for the methyl phosphoramidate modification of the heptose and Gal*f*NAc moieties. The green-colored genes are responsible for the biosynthesis of GDP-d-*glycero*-l-*gluco*-heptose. The blue-colored genes are responsible for the biosynthesis of UDP-GlcA and subsequent amidation with either serinol or ethanolamine. The genes colored yellow have an unknown function. The dark gray genes have been annotated as putative glycosyltransferases. Additional details are provided in the text.

Much is currently known about the chemical steps that facilitate the biosynthesis of the CPS in the HS:2 serotype of C. jejuni. Genetic and enzymatic experiments have shown that the enzymes Cj1415-Cj1418, Cj1421, and Cj1422 are primarily responsible for the phosphoramidation of the heptose and d-Gal*f*NAc moieties of the CPS.^20–23^ Cj1423-Cj1428, Cj1430, and Cj1431 are essential for the biosynthesis of GDP-d-*glycero*-l-*gluco*-heptose and the subsequent glycosylation of the d-glucuronamide moiety of the CPS.^24–33^ Enzymes Cj1435-Cj1437, Cj1441, and the C-terminal domain of Cj1438 are required for the amidation of the d-glucuronic acid moiety with either serinol or ethanolamine.^34–37^ Less is known about the identity and catalytic properties of the glycosyltransferases that are used to polymerize the activated carbohydrates into a repeating polymer of d-ribose, d-Gal*f*NAc, and d-GlcA. However, we have recently demonstrated that the N-terminal domain of Cj1432 is responsible for the transfer of d-glucuronic acid from UDP-GlcA to C2 of the d-riboside moiety at the nonreducing end of the growing CPS chain.^38^ We have also shown that the N-terminal domains of Cj1434 and Cj1438 catalyze the transfer of d-Gal*f*NAc to C4 of the d-glucuronamide moiety of the growing CPS chain.^39^ Here we demonstrate that the middle and C-terminal domains of Cj1432 are responsible for the transfer of d-ribose to C5 of d-Gal*f*NAc at the end of the growing polysaccharide chain.

## Materials and Methods

### Materials and Equipment

All materials and chemicals were obtained from Sigma-Aldrich, Biosynth, Carbosynth, GE Healthcare, or Research Products International, unless otherwise stated. Escherichia coli BL21 (DE3) cells were obtained from New England Biolabs. ArcticExpress (DE3) competent cells was purchased from Agilent. HisTrap columns, HiTrap Q HP anion exchange columns, Vivaspin 20 10 kDa MWCO or Vivaspin 6 10 kDa MWCO spin filters were obtained from Cytiva. The synthesis of methyl 2-acetamido-2-deoxy-β-d-galactofuranoside (**1**) is described in the Supporting Information. The enzymatic synthesis of disaccharide **7** was described previously.^39^ Ultraviolet spectra were collected on a SpectraMax ABS Plus UV–vis plate reader (Molecular Devices) using a 1 cm quartz cuvette. Nuclear magnetic resonance (NMR) spectra were recorded on a Bruker Avance III 400 MHz system equipped with a broadband probe and sample changer, or a Bruker Avance III 500 MHz NMR spectrometer. Mass spectrometry data were collected on a Thermo Scientific Q Exactive Focus system. The structures of the substrates and products isolated for this investigation are provided in Figure 3 and the calculated *m*/*z* values for the various products are listed in Table 1.

**Figure 3:**
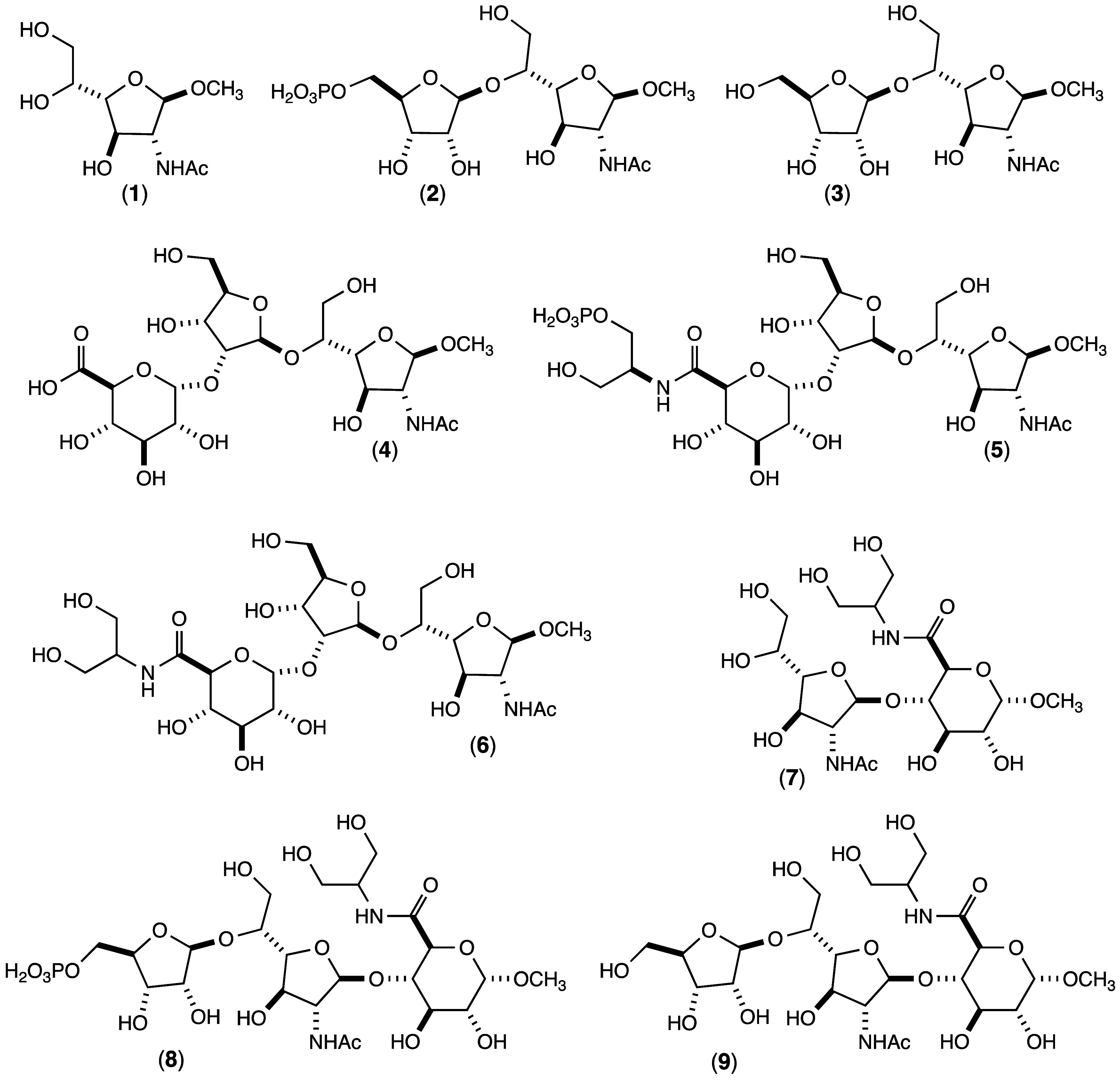
Structures of substrates and products utilized in this investigation.

**Table 1:** Calculated *m/z* Values for Disaccharide and Trisaccharide Reaction Products

product	exact mass	[M – H]^−^	[M + H]^+^	[M + Na]^+^	[M + K]^+^
**2**	447.11	446.11			
**3**	367.15		368.16	390.14	
**4**	543.18	542.17			
**5**	696.20	695.19			
**6**	616.23		617.24	639.22	655.20
**8**	696.20	695.19			
**9**	616.23		617.24	639.22	655.20

### Plasmid Construction

The gene encoding Cj1432 (Uniprot id: Q0P8I2) from C. jejuni NCTC 11168 genomic DNA (ATCC 700819D-5) was chemically synthesized (GenScript) with codon optimization for E. coli expression. The full-length gene for Cj1432 (absent the nucleotides for the C-terminal 117 amino acids) was cloned into the NdeI-5′ and *Bam*HI-3′ restriction sites of the pMAL-c5X expression vector to fuse the maltose-binding protein (MBP) at the N-terminus. A C-terminal hexa-histidine tag was also added to facilitate the purification of the protein. The protein is denoted as Cj1432_NMC_ for this investigation and the amino acid sequence of the purified protein, and the codon optimized gene sequence are shown in Figure S1.

### Purification of Cj1432_NMC_

ArcticExpress (DE3) competent cells were transformed with the plasmid encoding Cj1432 _NMC_. For transformation, 1.0 μL of 50 ng/μL plasmid DNA of Cj1432_NMC_ was added to 50 μL of ArcticExpress (DE3) competent cells. The cells containing the plasmid DNA were incubated on ice for 10 min before electroporation using a MicroPulser Electroporator (Bio-Rad). One mL of LB medium was then added to the cuvette, and the cells transferred to a 15 mL sterile Falcon tube. The cells were grown for 1 h at 37 °C and 10 μL of the cells were spread on an agar plate containing 100 μg/mL ampicillin and 20 μg/mL gentamicin. The plate was incubated at 37 °C overnight (16 h) and well-separated colonies were obtained. Single colonies were inoculated in 5.0 mL of LB medium supplemented with 100 μg/mL ampicillin and 20 μg/mL gentamicin. The cells were grown at 37 °C overnight while being shaken at 140 rpm. Each 5 mL starter culture was used to inoculate 1 L of LB medium supplemented with 100 μg/mL ampicillin and 20 μg/mL gentamicin. The cells were grown at 37 °C until the OD_600_ of the culture reached ∼0.6–0.7. The culture was kept at 4 °C for 30 min. Gene expression was induced by the addition of IPTG to a final concentration of 1.0 mM. The culture was subsequently incubated for 48 h at 14 °C while being shaken at 140 rpm. The cells were harvested by centrifugation at 7000*g* for 10 min at 4 °C, frozen in liquid N_2_, and stored at −80 °C. The protein was purified at 25 °C. In a typical purification, ∼20 g of frozen cell paste was resuspended in 200 mL of buffer A (50 mM HEPES, 500 mM NaCl, and 10 mM imidazole, pH 8.0) supplemented with 0.05 mg/mL protease inhibitor cocktail powder and 40 units/mL DNase I. The suspended cells were lysed by sonication (QSONICA Sonicator Ultrasonic Processor) in an ice bath, and the supernatant solution was collected after centrifugation at 10,000*g* for 30 min at 4 °C. The supernatant solution was filtered through a 0.45 μm cellulose syringe filter (VWR) and then loaded onto a prepacked 5 mL HisTrap column. The protein was eluted with a linear gradient of buffer B (50 mM HEPES, 500 mM NaCl, and 500 mM imidazole, pH 8.0). Fractions containing the desired protein, as identified by SDS–polyacrylamide gel electrophoresis (SDS–PAGE), were combined and concentrated to ∼10 mL in a 20 mL spin filter with a 10 kDa molecular weight cutoff (Cytiva). The imidazole was removed from the protein solution by dialysis using 2 cycles of 1.0 L of buffer C (50 mM HEPES, 250 mM NaCl, pH 8.0) at 4 °C. The protein was concentrated to ∼3 mg/mL, aliquoted, frozen in liquid N_2_, and stored at −80 °C. Typically, about 0.5–1.0 mg Cj1432_NMC_ was obtained from 1 L of cell culture.

### Determination of Protein Concentrations

The concentration of protein was determined spectrophotometrically using a computationally derived molar absorption coefficient at 280 nm.^40^ The molecular weight and the value of ε_280_ used for calculating the concentration of Cj1432_NMC_ with the MBP tag were 151,885 Da and 209,550 M^–1^ cm^–1^, respectively.^40^

### Expression and Purification of Other Proteins

Expression and purification of Cj1435, the N-terminal domain of Cj1432 (Cj1432_N_), the N-terminal domain of Cj1438 (Cj1438_N_) and the C-terminal domain of Cj1438 (Cj1438_C_) were reported previously.^35,38,39^ The amino acid sequences for the purified proteins are provided in Figure S1.

### Isolation of Disaccharide Product **2**

Disaccharide **2** was isolated from the reaction catalyzed by Cj1432_NMC_ and further identified using ESI-mass spectrometry and ^1^H NMR spectroscopy. A 1.0 mL reaction containing 0.5 μM Cj1432_NMC_, 5.0 mM compound **1**, 5.0 mM PRPP, and 5.0 mM MgCl_2_ was incubated in 50 mM NH_4_HCO_3_, pH 8.0, at 25 °C for 2.5 h. The reaction mixture was filtered using a 10K Nanosep spin filter (PALL) to remove the protein, diluted with H_2_O, and then loaded onto a 5 mL HiTrap Q HP anion exchange column connected to an NGC Chromatography System (BioRad). Disaccharide **2** was eluted from the column with a linear gradient of NH_4_HCO_3_, pH 8.0 (0–50% of 500 mM NH_4_HCO_3_). Fractions of 2.0 mL were collected and analyzed by negative ion ESI-MS. The fractions containing disaccharide **2** were combined, lyophilized once, and dissolved in D_2_O for mass spectrometry and NMR analysis.

### Isolation of Disaccharide Product **3**

Disaccharide **3** was obtained by dephosphorylation of product **2** using Cj1432_NMC_. A 1.0 mL reaction mixture containing 5.0 μM Cj1432_NMC_, 5.0 mM compound **1**, 5.0 mM PRPP, and 5.0 mM MgCl_2_ was incubated in 50 mM NH_4_HCO_3_, pH 8.0, at 25 °C for 30 min. Disaccharide **3** was obtained after removal of P_i_ and PP_i_ by anion exchange chromatography.

### Isolation of Trisaccharide Product **4**

Trisaccharide product **4** was made by the incubation of 50 μM Cj1432_N_ with 2.0 mM product **3**, 5.0 mM UDP-GlcA in 50 mM NH_4_HCO_3_, pH 8.0, at 25 °C for 18 h. The protein was removed using 10K Nanosep spin filters. The reaction mixture was loaded onto a 5 mL HiTrap Q HP anion exchange column connected to an F10 NGC Chromatography System and washed thoroughly with water. Trisaccharide product **4** was eluted from the column with a linear gradient of NH_4_HCO_3_ (0–50% of 500 mM NH_4_HCO_3_) and the individual fractions were analyzed using mass spectrometry. The fractions containing the desired product were pooled (6 mL total), lyophilized once to dryness, and dissolved in D_2_O for mass spectrometry and ^1^H NMR analysis.

### Isolation of Trisaccharide Product **5**

Product **5** was isolated from the reaction of 5.0 mM ATP, 5.0 mM (*S*)-serinol phosphate, 2.0 mM product **4**, 10 mM MgCl_2_, and 10 μM Cj1438_C_ in 50 mM NH_4_HCO_3_, pH 8.0.^36^ The reaction was incubated at 25 °C for 4 h and the protein was removed using a 10K Nanosep spin filter. The reaction mixture was then loaded onto a 5 mL HiTrap Q HP anion exchange column connected to an NGC Chromatography System and washed thoroughly with water. Reaction product **5** was eluted from the column with a gradient of NH_4_HCO_3_ (0–50% of 500 mM NH_4_HCO_3_) and each fraction (2.0 mL) was analyzed using mass spectrometry. The fractions containing trisaccharide product **5** were pooled (6.0 mL), lyophilized once to dryness, dissolved in D_2_O, and analyzed by mass spectrometry and ^1^H NMR spectroscopy.

### Isolation of Trisaccharide Product **6**

Product **6** was obtained by the dephosphorylation of product **5** using Cj1435.^35^ The reaction contained 5.0 μM Cj1435, 2.0 mM product **5**, and 50 mM NH_4_HCO_3_, pH 8.0, and was incubated at 25 °C for 4 h. The protein was removed using a 10K Nanosep spin filter. The reaction mixture was then loaded onto a 5 mL HiTrap Q HP anion exchange column connected to an NGC Chromatography System. The uncharged product **6** eluted from the column in the flowthrough. The fractions of the flowthrough were analyzed using mass spectrometry to identify product **6**.

### Isolation of Trisaccharide Product **8**

Trisaccharide product **8** was obtained from the reaction catalyzed by Cj1432_NMC_ using disaccharide **7** and PRPP as substrates.^39^ The 1.0 mL reaction mixture containing 0.5 μM Cj1432_NMC_, 5.0 mM compound **7**, 5.0 mM PRPP, 5.0 mM MgCl_2_, and 50 mM NH_4_HCO_3_, pH 8.0, was incubated at 25 °C for 4 h. The reaction mixture was filtered using a 10K Nanosep spin filter to remove the protein, diluted in H_2_O and then loaded onto a 5 mL HiTrap Q HP anion exchange column and washed thoroughly with water. The product was eluted with a gradient of NH_4_HCO_3_, pH 8.0 (0–50% of 500 mM NH_4_HCO_3_). The fractions were collected (2.0 mL each) and analyzed using mass spectrometry. The fractions containing trisaccharide **8** were combined (6.0 mL), lyophilized once, and dissolved in D_2_O for mass spectrometry and ^1^H NMR analysis.

### Isolation of Trisaccharide Product **9**

Product **9** was obtained by dephosphorylation of trisaccharide product **8** using Cj1432_NMC_. The 1.0 mL reaction mixture containing 10 μM Cj1432_NMC_, 5.0 mM product **8**, 5.0 mM PRPP, and 5.0 mM MgCl_2_ was incubated in 50 mM NH_4_HCO_3_, pH 8.0, at 25 °C for 1 h. Trisaccharide product **9** was obtained.

### Reaction Rate Determination

The rate of formation of product **2** from substrate **1** and PRPP, and the subsequent hydrolysis of P_i_ from product **2** was determined using ^31^P NMR spectroscopy. The reaction was initiated by the addition of 4.5 μM Cj1432_NMC_ to a 1.0 mL solution containing 5.0 mM compound **1**, 5.0 mM PRPP, and 5.0 mM MgCl_2_ in 50 mM HEPES, pH 8.0, at 30 °C. The reaction was monitored by following the consumption of PRPP at 3.82 ppm and the formation of P_i_ at 2.48 ppm as a function of time.

## Results and Discussion

### Expression and Purification of Cj1432_NMC_

The three-dimensional (3D) structure of full-length Cj1432 (1031 aa) was predicted using AlphaFold2 (Figure 4).^41^ From this structure it appears that Cj1432 is a three-domain protein with an additional four-helix bundle at the C-terminus. The functional significance of the four-helix bundle is unclear. This domain could be involved in protein–protein interactions, association with the inner membrane, or some other unknown function. We initially attempted to express and purify the middle domain of Cj1432 (Cj1432_M_; residues 357–573), the C-terminal domain of Cj1432 (Cj1432_C_; residues 574–907), and the combined central and C-terminal domains (Cj1432_MC_; residues 357–907) using a pET28a (+) vector with N-terminal hexahistidine tags. All three constructs expressed well in E. coli (DE3) cells, but the proteins were insoluble. Instead of slightly modifying our initial truncated protein designs we elected to make a construct for the expression of the entire Cj1432 protein (denoted here as Cj1432_NMC_) that lacks the C-terminal 117 amino acids into a pMAL-c5X vector with a maltose-binding protein (MBP) tag at the N-terminus and a hexa-histidine tag fused to the C-terminus. The enzyme Cj1432_NMC_ was expressed in ArcticExpress (DE3) cells and purified in relatively low yield with about 0.5–1.0 mg protein obtained from a 1.0 L culture.

**Figure 4:**
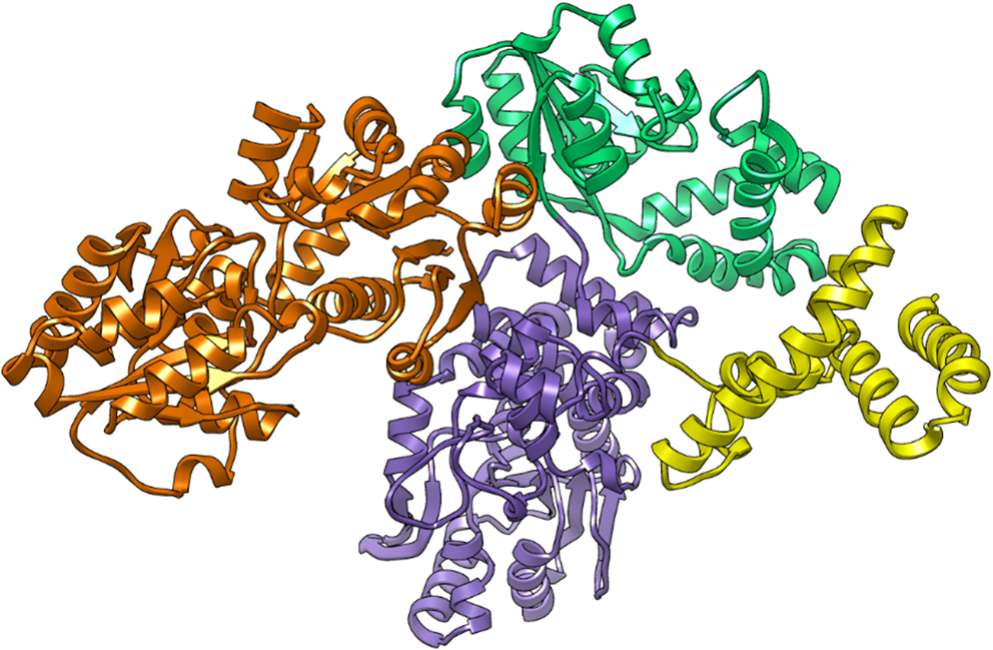
AlphaFold2 generated structure of Cj1432 (AF-Q0P8I2–F1-v4). The three-dimensional structure of the brown domain (residues 1–356) corresponds to a GT4 glycosyltransferase (denoted here as Cj1432_N_). The purple-colored domain (denoted here as Cj1432_C_; residues 574–914) is predicted to catalyze the transfer of d-ribose-5-P to C5 of the terminal d-Gal*f*NAc moiety of the growing polysaccharide chain and the green-colored domain (denoted here as Cj1432_M_; residues 357–573) is predicted to catalyze the hydrolysis of phosphate from this product. The terminal yellow domain (residues 915–1031) folds as a four α-helix bundle of unknown function. Additional details are provided in the text.

### Catalytic Activity of Cj1432_NMC_

The N-terminal domain of Cj1432 (Cj1432_N_) was previously identified as a GT4 glycosyltransferase for the transfer of D-GlcA to C2 of an acceptor d-riboside substrate with retention of configuration.^38^ Cj1432_N_ is highlighted in brown in Figure 4. The remaining two domains (colored green and purple) are predicted to be required for the transfer of d-ribose-5-P from PRPP to C5 of the terminal d-Gal*f*NAc moiety of the growing polysaccharide chain, and the subsequent hydrolysis of phosphate from the transient product. This prediction is based on the structural similarity of these two domains to an enzyme that was recently identified for the transfer of d-ribose-5-P from PRPP to the d-ribitol-5-P moiety in the CPS of Haemophilus influenzae.^42,43^

The catalytic activity of Cj1432_NMC_ was tested with the methyl glycoside acceptor **1** and PRPP in the presence of MgCl_2_. This multidomain enzyme was found to catalyze the initial formation of disaccharide **2** and subsequent phosphate hydrolysis to disaccharide **3** shown in Scheme 1. The reaction was monitored as a function of time using ^31^P NMR spectroscopy from −14 to 6 ppm (Figure S2). The initial transfer of d-ribose-5-P to acceptor **1** is catalyzed by the C-terminal domain (Cj1432_C_) and the hydrolysis of phosphate from this product is catalyzed by the middle HAD-phosphatase domain (Cj1432_M_). Under the reaction conditions of the initial experiment, the transfer of d-ribose-5-P from PRPP is faster than the subsequent hydrolysis of disaccharide **2** to disaccharide **3** as shown in Figures S2 and S3. Higher concentrations of Cj1432 or longer incubations make compound **3** the predominant reaction product. Disaccharide **2** was successfully isolated via anion exchange chromatography. The ESI-MS results for products **2** and **3** are shown in Figure 5a,b, respectively. An *m*/*z* of 446.10 appears in the mass spectrum for product **2** (Figure 5a). This peak corresponds to that expected for the [M – H]^−^ anion. An *m*/*z* of 368.15 and 390.13 appears in the mass spectrum for product **3** (Figure 5b). These two peaks correspond to that expected for the [M + H]^+^ and [M + Na]^+^ cations of product **3**, respectively. The initial rate of product **2** formation using 5.0 mM PRPP and 5.0 mM compound **1** as substrates was determined to be 37 ± 2 min^–1^ by following the change in the concentration of PRPP as a function of time using ^31^P NMR spectroscopy (Figure S3). The rate of phosphate hydrolysis from compound **2** was determined to be 3.4 ± 0.2 min^–1^ (Figure S3).

**Figure 5:**
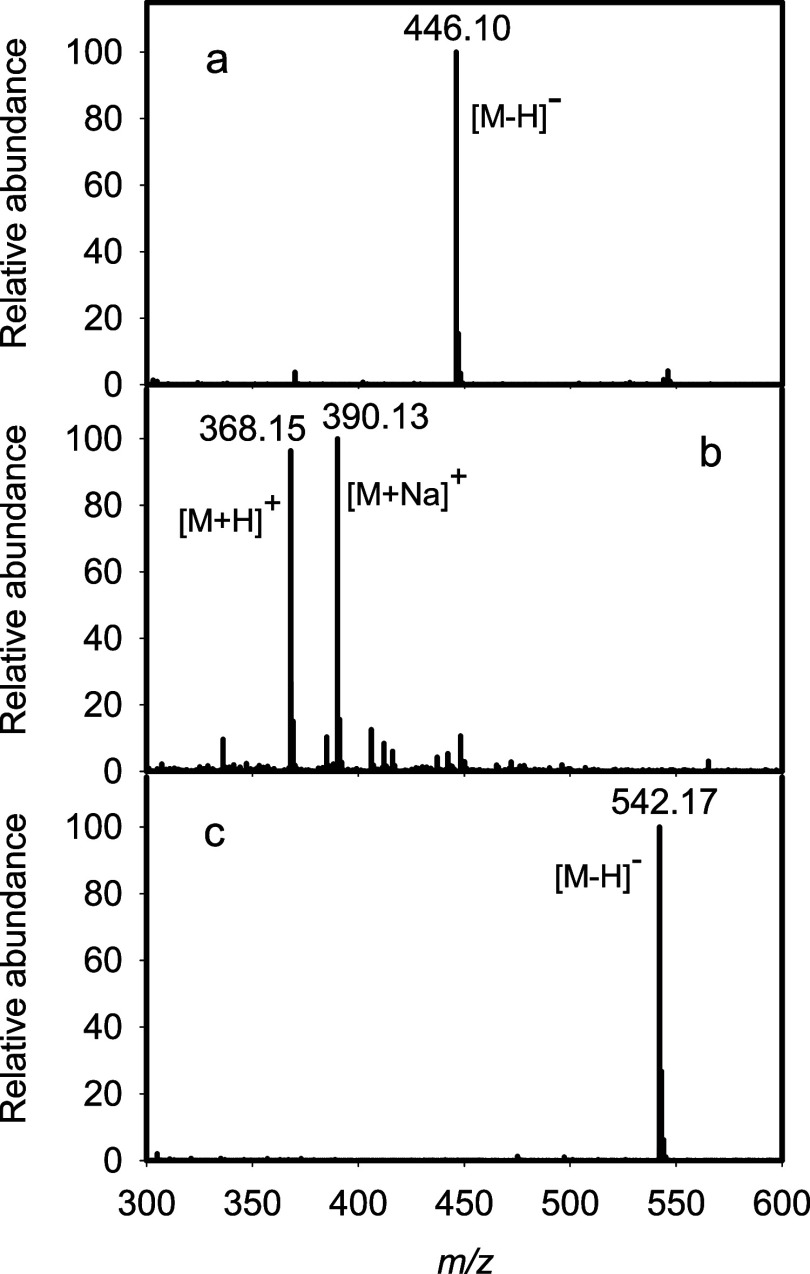
ESI mass spectrometry of products **2** (panel a), **3** (panel b), and **4** (panel c).

**Scheme 1:**
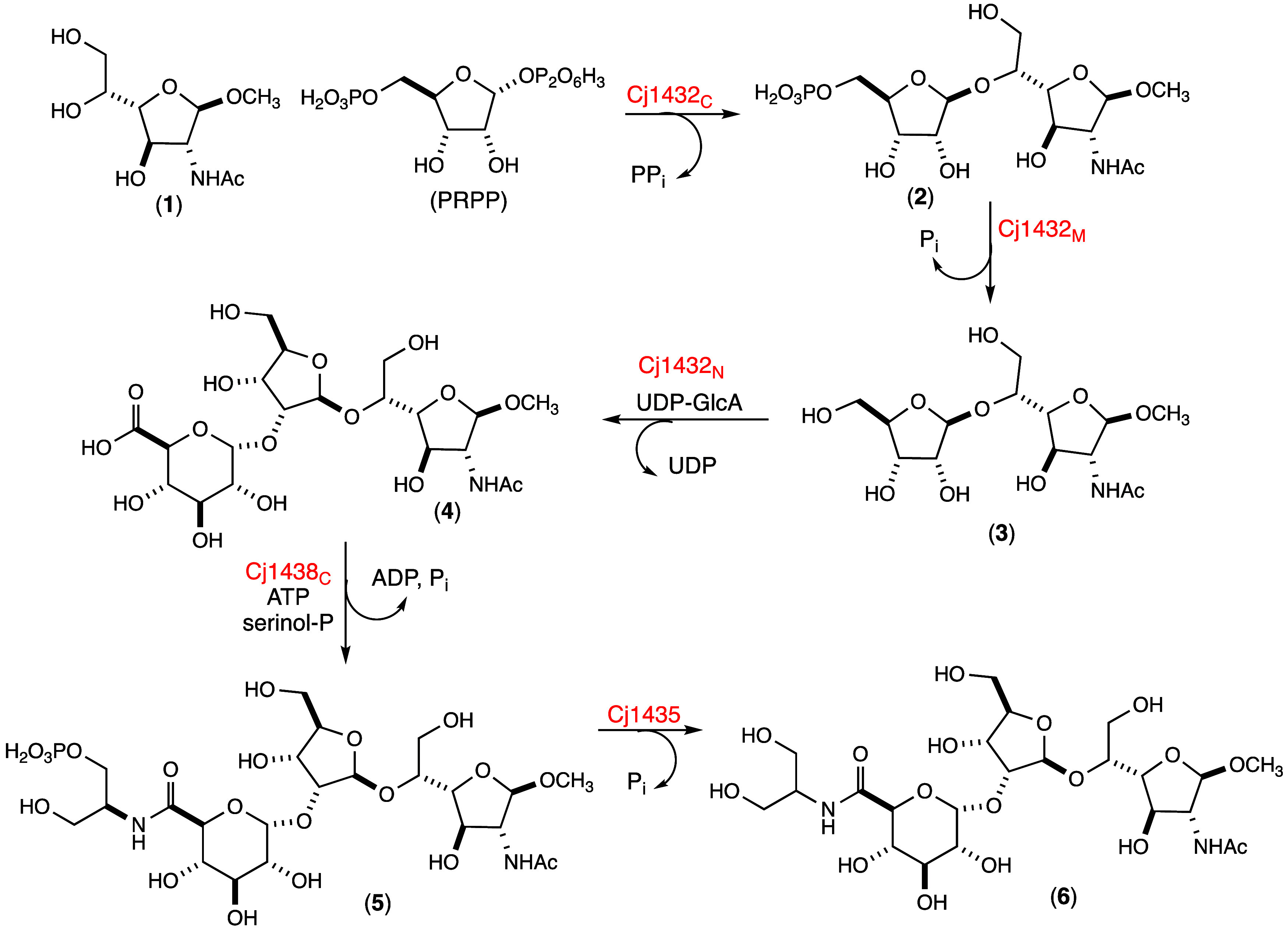
Reaction Scheme for the Synthesis of Products **2**, **3**, **4, 5**, and **6** Using Cj1432, Cj1438, and Cj1435

Disaccharide **2** was further analyzed using ^1^H NMR spectroscopy (Figure 6a). The resonances for the anomeric hydrogens of the d-ribose and the d-Gal*f*NAc moieties of product **2** appear at 4.85 and 5.24 ppm, respectively. The HSQC NMR spectra of substrate **1** and product **2** are presented in Figures S4 and S5, respectively. The downfield shift of ∼4.4 ppm for C5 of product **2** (75.2 ppm), relative to C5 of substrate **1** (70.8 ppm), is fully consistent with the attack of the hydroxyl group at C5 of substrate **1** with the anomeric carbon of PRPP as originally shown for the chemical characterization of the CPS from the HS:2 serotype of C. jejuni NCTC 11168.^18^

**Figure 6:**
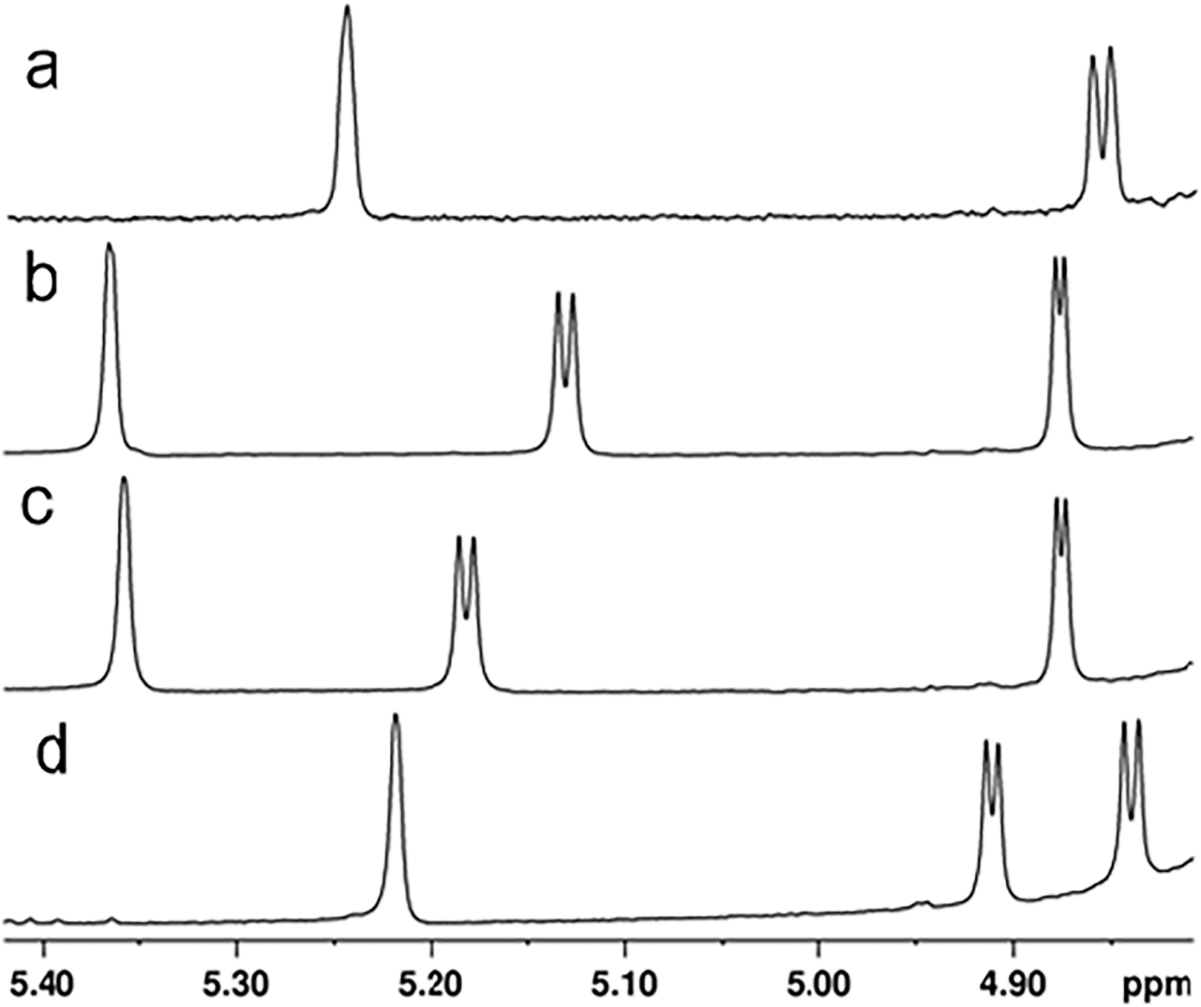
Portion of the ^1^H NMR spectrum of the Cj1432_NMC_ catalyzed reaction products showing the resonances for the anomeric hydrogens. (a) Disaccharide **2**; the singlet at ∼5.24 ppm originates from the d-Gal*f*NAc moiety and the doublet at ∼4.86 originates from the d-ribose moiety. (b) Product **4**; the singlet at ∼5.37 ppm originates from the d-Gal*f*NAc moiety, the doublet at ∼5.13 ppm originates from the d-GlcA moiety and the doublet at ∼4.88 ppm originates from the d-ribose moiety. (c) Product **5;** the singlet at 5.36 ppm originates from the d-Gal*f*NAc moiety, the doublet at 5.18 ppm originates from the d-GlcA moiety, and the doublet at 4.82 ppm originates from the d-ribose moiety. (d) Product **8**; the singlet at 5.22 ppm originates from the d-Gal*f*NAc moiety, the doublet at 4.91 ppm originates from the d-ribose moiety, and the doublet at 4.84 ppm originates from the d-GlcA moiety.

Disaccharide product **3** was used as the acceptor substrate with UDP-GlcA as the donor substrate in the presence of either Cj1432_N_ or Cj1432_NMC_ to synthesize trisaccharide **4** as illustrated in Scheme 1. Product **4** was purified using anion exchange chromatography and structurally identified using mass spectrometry and ^1^H NMR spectroscopy. An *m*/*z* of 542.17 appears in the mass spectrum of product **4** for the [M – H]^−^ anion (Figure 5c). The portion of the ^1^H NMR spectrum for the anomeric hydrogens of trisaccharide **4** is shown in Figure 6b. The resonances for anomeric hydrogens of d-ribose, d-GlcA, and d-Gal*f*NAc moieties of **4** appear at 4.87, 5.13, and 5.36 ppm, respectively. The full ^1^H NMR and ^1^H–^13^C HSQC spectra for compound **4** are presented in Figure S6.

### Synthesis of Trisaccharide Products **5** and **6**

Previously, the C-terminal domain of Cj1438 (Cj1438_C_) was demonstrated to catalyze amide bond formation using the C6-carboxylate of substrates containing a terminal D-GlcA moiety in the presence of MgATP and (*S*)-serinol phosphate (or ethanolamine phosphate).^36^ Therefore, trisaccharide product **4** was used to make product **5** by the catalytic activity of Cj1438_C_ (Scheme 1). Product **5** was purified using anion exchange chromatography and structurally characterized by ESI mass spectrometry and ^1^H NMR spectroscopy. The ESI-MS for trisaccharide **5** is presented in Figure 7a. An *m*/*z* of 695.19 is detected for the [M-H]^−^ anion. The full ^1^H NMR and ^1^H–^13^C HSQC spectra for compound **5** are presented in Figure 7 and the portion of the ^1^H NMR spectrum for the anomeric hydrogens of trisaccharide **5** is shown in Figure 6c.

**Figure 7:**
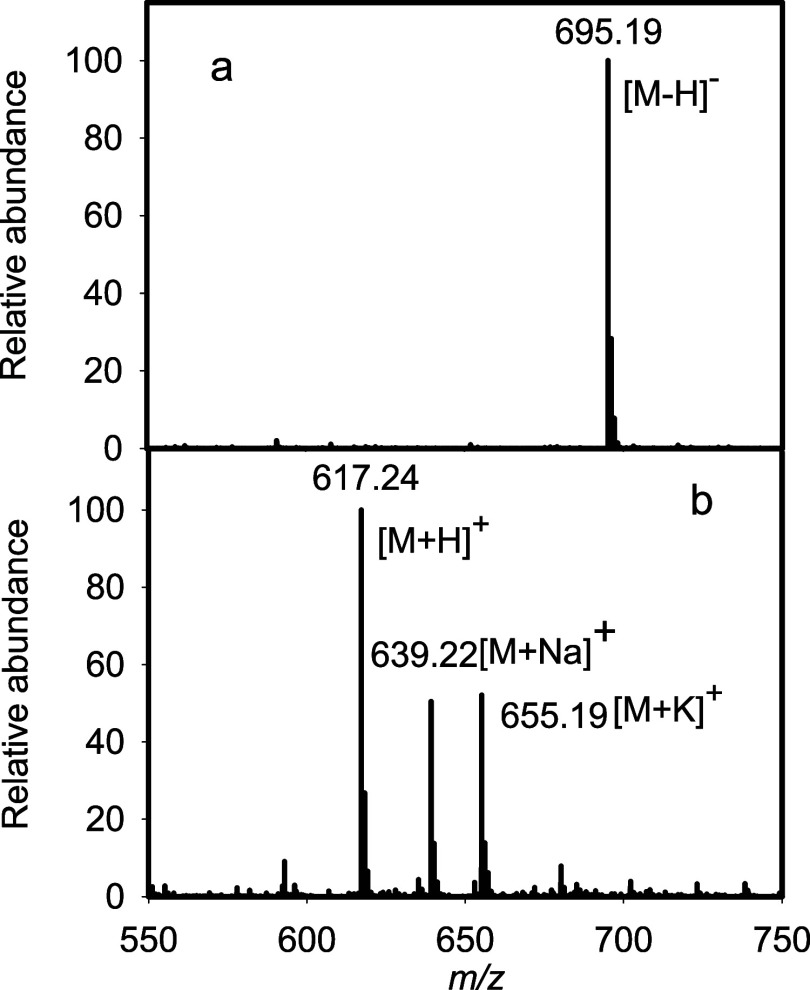
ESI mass spectra of products **5** (panel a) and **6** (panel **b**).

Trisaccharide **6** was obtained by dephosphorylation of product **5** as catalyzed by Cj1435.^35^ Product **6** was purified using the HiTrap Q HP anion exchange column to remove any of the other anionic products and subsequently analyzed using ESI mass spectrometry. The *m*/*z* values of 617.24, 639.22, and 655.19 appear in the mass spectrum for the [M + H]^+^, [M + Na]^+^, and [M + K]^+^ cations of product **6**, respectively. The entire reaction pathway for the conversion of the methyl glycoside of d-Gal*f*NAc (**1**) to trisaccharide (**6**) is summarized in Scheme 1.

### Synthesis of Trisaccharide Products **8** and **9**

The catalytic activity of Cj1432_NMC_ was also used to synthesize two additional trisaccharide products (compounds **8** and **9**). When disaccharide **7** was used as the initial accepting sugar in the presence of PRPP and Cj1432_NMC_, trisaccharide product **8** was obtained. Product **8** was purified via anion exchange chromatography and structurally characterized using ESI-MS (Figure 8a) and ^1^H NMR spectroscopy (Figure 6c). An *m*/*z* of 695.19 is detected for the [M – H]^−^ anion in the mass spectrum. The resonances for anomeric hydrogens of the d-ribose, d-GlcA, and d-Gal*f*NAc moieties of product **8** appear at 4.91, 4.84, and 5.22 ppm, respectively. The full ^1^H NMR and HSQC spectra for product **8** are presented in Figure S8.

**Figure 8:**
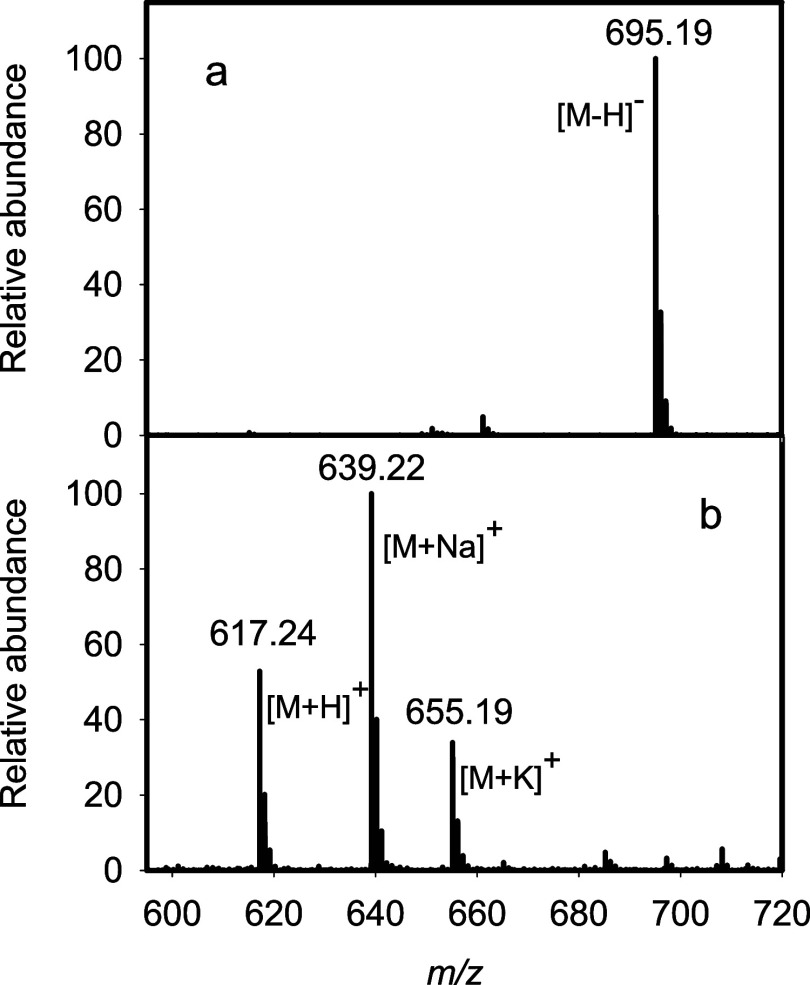
ESI-MS of trisaccharide products **8** (panel a) and **9** (panel b).

Product **9** was obtained by dephosphorylation of product **8** catalyzed by the middle catalytic domain of Cj1432_NMC_. The dephosphorylated product **9** was purified using anion exchange chromatography to remove the phosphate product and subsequently analyzed using ESI mass spectrometry (Figure 8b) which provided *m*/*z* values of 617.24, 639.22, and 655.19 for the [M + H]^+^, [M + Na]^+^, and [M+K]^+^ cations, respectively. The reactions are summarized in Scheme 2.

**Scheme 2:**
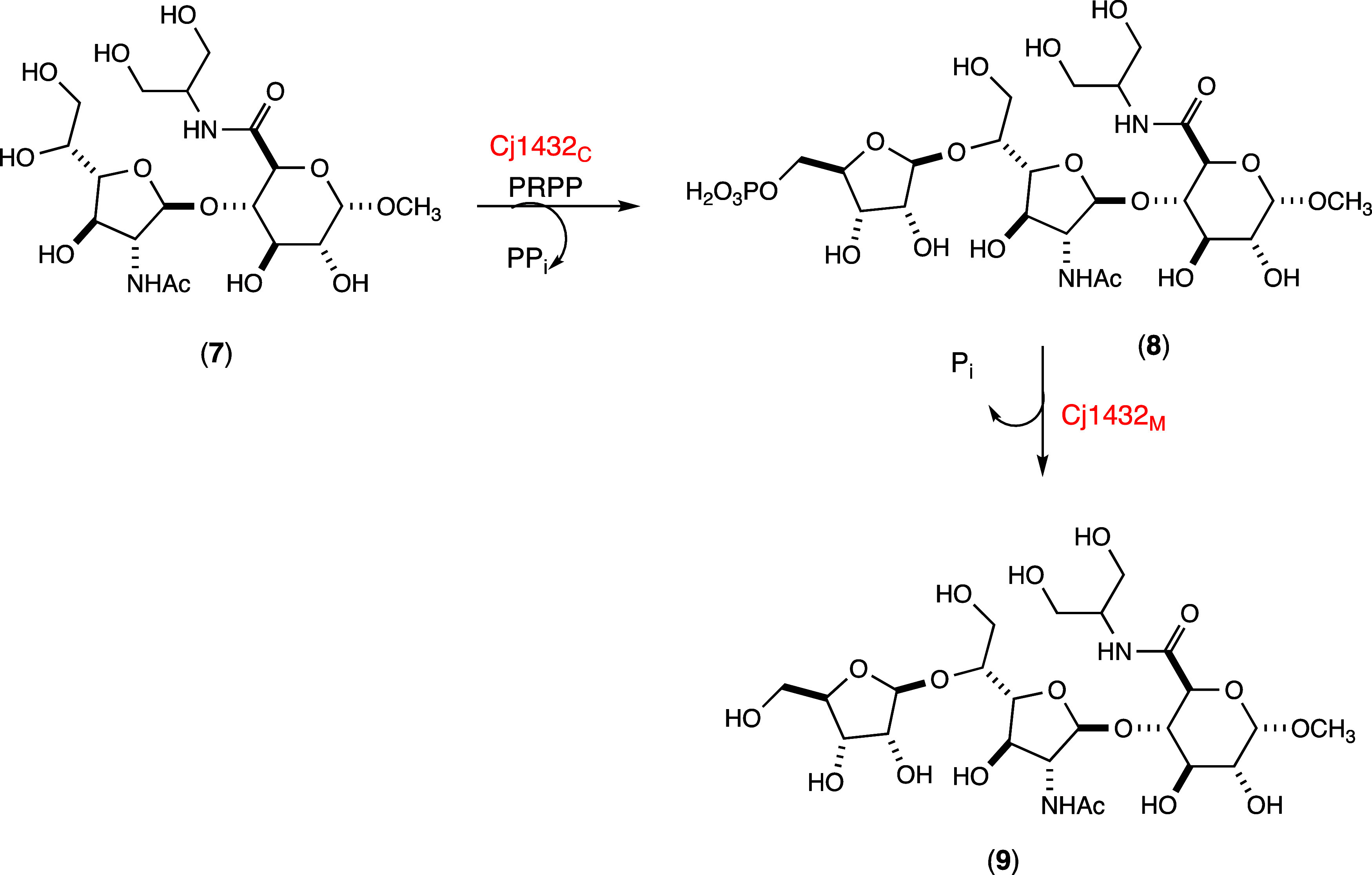
Reaction Scheme for the Enzyme-Catalyzed Synthesis of Trisaccharides **8** and **9**

Here we have demonstrated for the first time that Cj1432 is a multienzyme assembly, which catalyzes three different reactions during the biosynthesis of the CPS of C. jejuni. The C-terminal domain of Cj1432 (Cj1432_C_; residues 574–914) catalyzes the transfer of ribose-5-P from PRPP to C5 of the Gal*f*NAc moiety at the growing end of the polysaccharide chain. The product of this reaction is dephosphorylated by the catalytic activity of the middle domain of Cj1432 (Cj1432_M_; residue 357–573). The third reaction is catalyzed by the N-terminal domain (Cj1432_N_: residues 1–356) where UDP-GlcA is used to transfer GlcA to the C2 hydroxyl group of the d-ribose moiety at the nonreducing end of the growing polysaccharide chain.^38,39^

Recently, a multidomain enzyme that catalyzes the polymerization of the type b capsule from H. influenzae was reported.^42^ This multifunctional enzyme contains an N-terminal ribitol-phosphate transferase domain (CroT), a central phosphatase domain (CrpP), and a C-terminal ribofuranosyltransferase domain (CriT). The N-terminal domain of Cj1432 (residues 1–356) and Crot (1–373) share no sequence similarity. The C-terminal domain of Cj1432 (residues 574–907) and CriT (residues 592–1036) have a sequence identity of 25% and the AlphaFold2 structure of the C-terminal domain of Cj1432 (Cj1432_C_) and the three-dimensional structure of CriT are quite similar to one another (Figure S9b). This can be explained by the common usage of PRPP to transfer d-ribose 5-P to an acceptor substrate. The central domain of Cj1432 (Cj1432_M_: residues 368–574) has a 31% sequence identity with CrpP and both domains catalyze the dephosphorylation of the terminal ribose-5P moiety (Figure S9a). The amino acid sequence alignments between CrpP and Cj1432_M_, and between CriT and Cj1432_C_ are shown in Figure S10a,b, respectively. A dual domain ribofuranosyltransferase from Klebsiella pneumoniae has also reported for the transfer of ribose-5-P from PRPP to C4 of a d-galactose acceptor and the dephosphorylation of the resulting product.^43^

Multifunctional enzyme clusters have been identified in the synthesis of other capsular polysaccharides. For example, we have previously shown that the C-terminal domain of Cj1438 from C. jejuni catalyzes ATP-dependent amide bond formation using the glucuronic acid moiety at the nonreducing end of the growing CPS with either serinol-phosphate or ethanolamine phosphate and that the N-terminal domain catalyzes the transfer of Gal*f*NAc from UDP-Gal*f*NAc.^38^ Recently, we also demonstrated that the multidomain enzyme HS1.09 from the HS:1 serotype of C. jejuni catalyzes the polymerization of d-galactose and glycerol-3-phosphate.^44^ This enzyme is similar in sequence and structure (PDB id: 8QOY) with Cps3D from Actinobacillus pleuropneumoniae, which also catalyzes the polymerization of d-galactose and glycerol-3-P during the biosynthesis of the CPS in this organism.^45^ Therefore, it appears that these multifunctional architectures are used repeatedly in the biosynthesis of capsular polysaccharides in bacteria.

## Conclusions

The minimal repeating polysaccharide unit that can be synthesized by the HS:2 serotype of C. jejuni consists of d-Rib, d-Gal*f*NAc, and the serinol amide of d-GlcA.^46^ Here we have demonstrated that the C-terminal domain of Cj1432 is responsible for the transfer of ribose-5-P from PRPP to C5 of the Gal*f*NAc moiety of the growing polysaccharide chain. In the next step, the middle domain of Cj1432 catalyzes the hydrolysis of phosphate from this product. We have shown previously that the N-terminal domain of Cj1432 catalyzes the transfer of GlcA from UDP-GlcA to C2 of the d-ribose moiety from the previous product and thus Cj1432 catalyzes three consecutive reactions during the biosynthesis of the capsular polysaccharide of C. jejuni.^38^ The product of the reactions catalyzed by Cj1432 is the substrate for the C-terminal domain of Cj1438, which catalyzes the ATP-dependent amidation of the C6-carboxylate of the terminal d-GlcA moiety of the growing polysaccharide chain with (*S*)-serinol phosphate.^38^ The phosphate of this product is hydrolyzed by the catalytic activity of Cj1435.^35,38^ In the last step of the cycle, the N-terminal domain of Cj1438 (or Cj1434) catalyzes the transfer of Gal*f*NAc from UDP-Gal*f*NAc to C4 of the terminal d-glucuronamide moiety.^39^ Therefore, to catalyze formation of the minimal repeating trisaccharide of the CPS of C. jejuni NCTC 11168 a total of six enzyme reactions are required with six intermediate structures. In addition to these six reactions, three additional reactions are required for the biosynthesis of UDP-GlcA from UDP-Glc (Cj1441), the transamination of DHAP with l-glutamate (Cj1437), and the conversion of UDP-Gal*p*NAc to UDP-Gal*f*NAc (Cj1439).^34–37,47^ This unprecedented set of nine enzymatic transformations is summarized in Scheme 3. Experiments designed to synthesize larger oligosaccharides composed of the repeating unit of d-ribose, d-GalfNAc, and the amide of d-glucuronate using the biosynthetic pathway outlined in Scheme 3 are currently in progress.

**Scheme 3:**
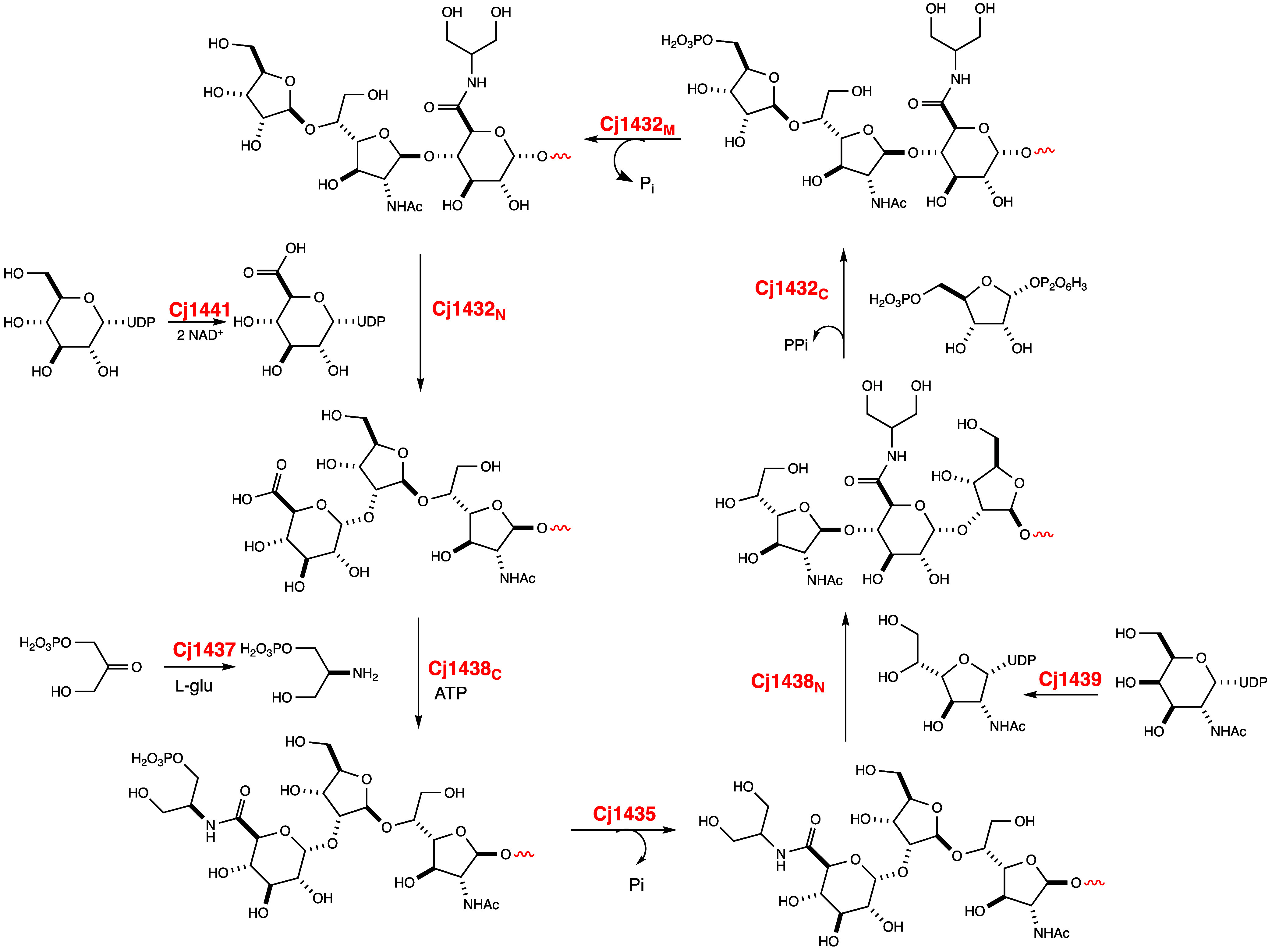
Biosynthetic Transformation for the Assembly of the Repeating Unit within the Capsular Polysaccharide of C. jejuni NCTC 11168 (Serotype HS:2)

## Supplementary Material



## Notes

Amino acid sequences of the purified proteins, and NMR spectra of chemically synthesized substrates and enzyme-catalyzed reaction products (PDF)
