# Supplementary material for: Identification and Functional Characterization of the Polymerizing Glycosyltransferase Required for the Transfer of d‑Ribose to the d‑GalfNAc Moiety of the Capsular Polysaccharide of Campylobacter jejuni
Source: Biochemistry. 2025 May 1;64(10):2226–36. doi: 10.1021/acs.biochem.5c00052 (PMC12096443; doi:10.1021/acs.biochem.5c00052)
Supplement: Supplementary file 1 [file bi5c00052_si_001.pdf]

Supplementary Information

Identification and Functional Characterization of the Polymerizing Glycosyltransferase Required for the Transfer of D-Ribose to the D-Gal*f*NAc Moiety of the Capsular Polysaccharide of *Campylobacter jejuni*

Dao Feng Xiang[ϕ], Tamari Narindoshvili[ϕ] , and Frank M. Raushel[ϕ,*]

[ϕ]Department of Chemistry, Texas A&M University, College Station, Texas, 77843, United States.

*Contact Information

e-mail: raushel@tamu.edu
phone: 1-979-845-3373

(a)

MKIEEGKLVIWINGDKGYNGLAEVGKKFEKDTGIKVTVEHPDKLEEKFPQVAATGDGPDIIFWAHDRFGGYAQSGLL
AEITPDKAFQDKLYPFTWDAVRYNGKLIAYPIAVEALSLIYNKDLLPNPPKTWEEIPALDKELKAKGKSALMFNLQE
PYFTWPLIAADGGYAFKYENGKYDIKDVGVDNAGAKAGLTFLVDLIKNKHMNADTDYSIAEAAFNKGETAMTINGPW
AWSNIDTSKVNYGVTVLPTFKGQPSKPFVGVLSAGINAASPNKELAKEFLENYLLTDEGLEAVNKDKPLGAVALKSY
EEELVKDPRIAATMENAQKGEIMPNIPQMSAFWYAVRTAVINAASGRQTVDEALKDAQTNSSSNNNNNNNNNNLGIE
GRISHMIKKILLITPELEYTGALNSFKRICEVLLNNKYAVDIWTYNEGPYISEFDKLGVYVEVISEDDIDSKWVHER
ISKYSLVIANTIVVYKCVELIQNLTPVVWYIREAENLPDFFWKPERKLALEKAKKLYVVSEYAKDFIIHNYNKNVEV
LHNYVDDVFYEKHDDFLKQIKSDKLKFLALGTIEKRKGYDVLLQAFIDLPVDIRDQCELHFAGRFWEGAKDFFPKIL
SLAKKFPNIFYHGELRDRKKIHSLIFQCNVMVVPSRDESCSLVALEGAMMSKPLILTENIGAKYILDENSGWLVKTG
SVDSLKNAFIQAYKNKNKLDAMGANSRNNYLQTSTYEIYEKNILKMVRDEICKNQYLYRINQENYVLFSFDIFDTLI
SRNIAKPSAVFLIMKQKMRNMDFPLNLVKNFDRIRVEVEQYYYRNVCKNKYEDTNFDEIYNLLQQNFSLSFQQKEEL
MKLEINTEKETLYPIKKNIELVEELIKNEKRVVLISDMYFSSSIIRTFLNKFSPIFNNIPIYMSSEFRLKKNSGNLF
KAILNLEKVDPKKWIHCGDNWVGDYLKPSNLEISTNFYINQLLPYEEFALNRNSLDMDLQKIIGISKKIRLENTLTN
LQEIGVSFGAPMLLPYVQWILNIALKNSIRCLYFIARDGYVLQKMTDMLIQAKKINIKTKYLYGSRESWREPFRNKD
KLKIQLIDEYLDQEIDKQEIFAFVECCGTGETLDYIVKRIESNQQFKNMFFGSLYLYRSKLNKTKTQSLFMLPLNEN
YTYGIELFVRSLQGQVLGYDKKDGRVIPVFDFLEGEALQKFRYDEYIDGVMLFMEYIVKTDNYEKIFDNMNVTILYL
NYLSNNYIDKKFIEIMGNVPFILNGVKDRVGIFAPRLNNKITLDQKNSFFNWSVLRSCKDIRVKYNMDNDCYSSENL
YFQGSSHHHHHH

(b)

MGSSHHHHHHSSENLYFQGHMIKKILLITPELEYTGALNSFKRICEVLLNNKYAVDIWTYNEGPYISEFDKLGVYVE
VISEDDIDSKWVHERISKYSLVIANTIVVYKCVELIQNLTPVVWYIREAENLPDFFWKPERKLALEKAKKLYVVSEY
AKDFIIHNYNKNVEVLHNYVDDVFYEKHDDFLKQIKSDKLKFLALGTIEKRKGYDVLLQAFIDLPVDIRDQCELHFA
GRFWEGAKDFFPKILSLAKKFPNIFYHGELRDRKKIHSLIFQCNVMVVPSRDESCSLVALEGAMMSKPLILTENIGA
KYILDENSGWLVKTGSVDSLKNAFIQAYKNKNKLDAMGANSRNNYLQTSTYEIYEKNILKMVRDEICK

(c)

MKELSDYDFLLNRHKQIFDYTPDFKCPVTFNEKLIYRILYDRSCIYSFLADKIKMRFYVASALSDNHEYSWDKIDIL
NEKSILFNNIDDLQDKIFETNKCKYLPKIYGIYKNIYDINFNELPNSFVLKTNHDCGGYVIVENKQEFLRDTVVFSN
AMKKLKKHLEWNYYSVFREWHYKDIEPRVFAEELLLGENKKPADTYKFHIFDKENLSNNFIQVTTDRFDNYQRAMFD
LSWNLAPFNFMYDNKNVTMIPKKPNLLDSMINISLILAKPFDYVRVDLYQFDKKIYIGELTFTHGAAGEKVIPKEWD
KKLGDLWRLKRLDNASKLEHHHHHH

(d)

MSKEILALFDFCETLTNFQTLDRYLPLAGSKNINYTQSKNLARRERFQRENLPYPRYEWLIDLDVDLAEEIAQEFVY
TDVMANLNQNVMDRLFWHQDEGHTIVIVSGGLTIYIKEFARIYNIENIVAVDLEIYKNKLTGNIDGIHTMQERKLYK
LAQKFNLKQFDLKNSYAYSDCVSDIPLLSLVGNPNVIECGKDLQWARILGFNILLKYLEHHHHHH

(e)

ATGATAAAGAAAATACTATTAATTACACCCGAACTGGAGTACACCGGTGCCCTGAACAGCTTCAAGCGCATCTGCGA
AGTCTTATTGAACAACAAATATGCGGTGGATATCTGGACCTACAATGAAGGTCCGTACATTTCGGAGTTTGATAAGC
TGGGTGTTTACGTTGAGGTCATTTCCGAGGACGACATTGATAGCAAATGGGTTCATGAGCGAATTAGCAAATATAGT
CTAGTGATCGCGAATACGATTGTGGTCTACAAATGTGTTGAGTTGATTCAGAACCTGACCCCAGTTGTTTGGTATAT
TCGTGAAGCGGAAAACCTGCCGGATTTCTTCTGGAAACCGGAACGTAAACTGGCTTTGGAGAAGGCGAAGAAGCTTT
ACGTCGTGAGCGAATACGCCAAAGACTTTATCATTCATAATTACAATAAGAACGTGGAGGTGCTGCACAACTACGTT
GACGATGTTTTTTACGAAAAGCACGATGATTTCCTGAAGCAGATTAAAAGCGATAAACTGAAGTTTCTGGCGCTGGG
CACCATTGAAAAGAGGAAAGGTTACGACGTACTGCTCCAAGCGTTTATCGACCTGCCGGTGGATATTCGTGATCAAT
GCGAACTTCACTTTGCTGGCCGTTTTTGGGAAGGCGCTAAGGACTTTTTTCCGAAAATTTTGAGCCTGGCGAAAAAA
TTCCCGAATATTTTTTATCATGGTGAACTGCGCGATCGTAAAAAGATCCACAGCTTGATCTTCCAGTGCAATGTGAT
GGTTGTGCCGTCTCGTGATGAGAGTTGCAGCCTTGTGGCATTGGAAGGCGCTATGATGAGCAAGCCGCTGATCCTGA
CTGAAAACATTGGCGCGAAGTATATCCTGGACGAGAACTCGGGTTGGTTGGTTAAGACTGGTTCCGTGGACAGCCTA
AAGAACGCCTTCATCCAGGCGTATAAAAATAAAAACAAGCTTGACGCAATGGGCGCGAATAGCCGCAACAATTATCT
GCAAACCTCAACCTACGAGATCTATGAAAAGAATATCTTAAAGATGGTTCGTGACGAGATCTGTAAAAACCAATACC
TGTACCGCATTAATCAAGAAAACTATGTCTTGTTCAGCTTTGATATCTTCGACACTCTGATCTCGCGTAACATCGCG
AAGCCTTCTGCAGTTTTTCTGATCATGAAACAGAAGATGCGTAATATGGACTTCCCGCTGAACCTGGTGAAAAACTT
CGACCGCATCCGTGTTGAGGTTGAACAGTATTACTATCGTAACGTCTGCAAAAATAAATACGAGGATACCAACTTCG
ACGAGATCTACAATCTGCTCCAACAAAACTTTTCTCTGAGCTTCCAGCAGAAAGAAGAGCTGATGAAATTGGAGATT
AACACCGAAAAAGAAACCCTGTACCCGATTAAGAAGAACATCGAGCTGGTGGAAGAACTCATCAAGAACGAAAAGCG
TGTGGTGCTGATTTCCGATATGTATTTTAGCTCCTCTATTATCCGCACCTTTCTCAACAAGTTCTCCCCGATTTTCA
ACAATATTCCGATCTATATGAGCTCCGAGTTCCGCCTGAAAAAAAACTCTGGCAATCTGTTCAAAGCGATTCTGAAC
CTGGAGAAAGTTGATCCGAAAAAGTGGATTCATTGTGGTGATAATTGGGTTGGTGATTATCTCAAACCAAGCAATTT
GGAGATCTCTACGAATTTCTATATCAACCAGCTGCTGCCGTATGAAGAATTCGCCTTAAATCGTAATTCCCTTGATA
TGGATCTGCAGAAGATCATTGGCATTTCCAAGAAGATCCGGCTGGAAAACACCCTGACGAACTTGCAGGAGATTGGC
GTGTCCTTTGGTGCTCCGATGCTGCTCCCTTACGTACAGTGGATTCTGAACATCGCGCTGAAGAACTCGATTCGCTG
CCTATATTTCATCGCGCGTGATGGTTATGTTCTGCAAAAAATGACCGATATGCTGATTCAAGCCAAGAAAATCAATA
TCAAGACCAAATACCTGTACGGCAGCCGTGAGAGCTGGCGTGAACCGTTTCGCAATAAGGACAAACTTAAAATCCAA
CTGATCGACGAGTACTTGGACCAAGAGATCGACAAGCAGGAGATCTTCGCGTTTGTCGAGTGCTGCGGTACAGGTGA
AACGCTGGACTATATCGTGAAAAGAATCGAAAGCAACCAGCAGTTTAAAAACATGTTTTTCGGCAGCCTGTATCTGT
ACCGTAGCAAGTTGAACAAAACGAAAACCCAGAGCCTGTTTATGTTACCGCTGAACGAGAACTACACCTATGGTATT
GAACTGTTCGTTCGTTCTCTCCAAGGTCAGGTTCTGGGTTACGATAAGAAGGACGGCCGTGTCATTCCGGTTTTCGA
CTTCCTGGAGGGCGAAGCATTGCAGAAATTTCGCTATGATGAGTATATCGACGGCGTAATGCTGTTCATGGAATACA
TTGTGAAAACCGATAACTATGAGAAGATCTTCGACAACATGAACGTGACCATCCTGTATCTGAACTACCTCTCTAAC
AACTACATTGACAAAAAATTCATCGAAATCATGGGTAATGTGCCGTTTATCTTAAATGGGGTTAAGGACCGCGTGGG
CATTTTCGCACCGCGTCTGAATAACAAGATTACCTTGGACCAAAAGAATTCCTTCTTTAACTGGAGCGTTTTGCGTT
CATGCAAAGACATACGCGTGAAGTACAACATGGACAATGATTGTTATAGCAGCGAGAACTTGTACTTTCAAGGTAGC
AGCCATCACCACCATCACCACTAA

Figure S1: Amino acid sequences of the proteins used for this investigation and the codon-optimized gene sequence used for $Cj1432_{1\text{-}914}$. (a) MBP-$Cj1432_{1\text{-}914}$ ($Cj1432_{NMC}$). The sequence of MBP tag is colored in green, the polyhistidine tag at the C-terminal end is shown in red font and the linker inserted between the MBP and Cj1432 is shown in orange. (b) Amino acid sequence for $Cj1432_{N}$.[1] The polyhistidine tag at the N-terminal end is shown in red font. (c) Amino acid sequence for $Cj1438_{C}$.[2] The added polyhistidine purification tag at the C-terminal end is shown in red font. (d) The amino acid sequence for Cj1435.[3] The added polyhistidine purification tag at the C-terminal end of the protein is shown in red font. (e) codon-optimized gene sequence used for $Cj1432_{1\text{-}914.}$

Synthesis of Methyl 2-acetamido-2-deoxy-β-D-galactofuranoside (1).

D-Galactosamine hydrochloride (**S1**) was converted into 2-azido-2-deoxy-D-galactose (**S2)** by freshly prepared triflyl azide in catalytic diazotransfer reaction according to the described procedure.[4] Synthesis of the α and β (1:3) mixture of methyl 3,5,6-tri-O-acetyl-2-azido-2-deoxy-D-galactofuranoside (**S3)** was achieved according to literature reports.[5] The desired β anomer (**S3a)** was isolated by column chromatography (hexanes: EtOAc, 3:1). **S3a** was subjected to one-pot azido group reduction and subsequent acetylation reactions to produce **S4** and the acetyl groups were removed to obtain desired product **1**. Procedures for the conversions were performed as reported.[5]

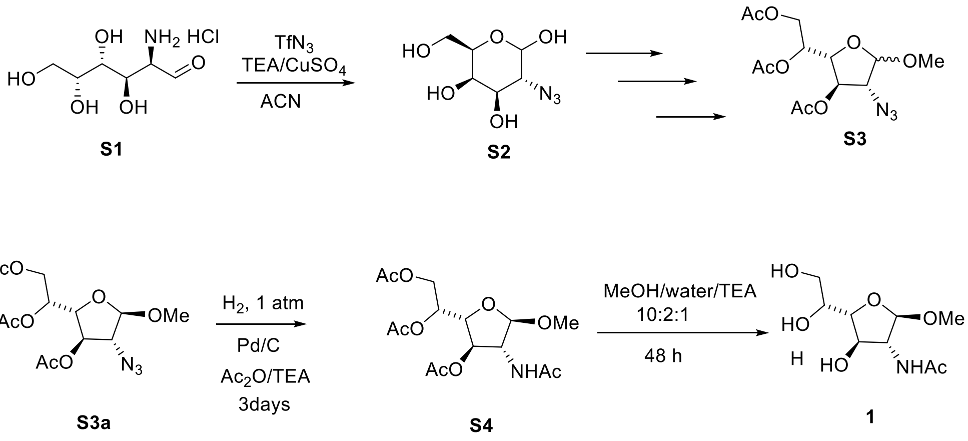

Scheme S1: Synthesis of methyl 2-acetamido-2-deoxy-β-D-galactofuranoside (**1**).

Methyl 2-acetamido-2-deoxy-β-D-galactofuranoside (**1**):

^{1}H NMR (400 MHz, D_2O) δ 4.90 (s, 1H), 4.16-4.10 (m, 2H), 4.02-3.97 (m, 1H), 3.87-3.80 (m, 1H), 3.71 (dd, J_1 = 4.8 Hz, J_2 = 11.6 Hz, 1H), 3.65 (dd, J_1 = 7.2 Hz, J_2 = 11.6 Hz, 1H), 3.40 (s, 3H), 2.01 (s, 3H) ppm.

^{13}C NMR (125 MHz, MeOH-*d*$_4$) δ 171.6, 107.6, 83.1, 76.2, 70.8, 63.1, 62.9, 53.8, 21.3. HRMS (ESI$^+$) *m/z* [M + H]$^+$ calc. for $C_9H_{18}NO_6$: 236.1134, found: 236.1127.

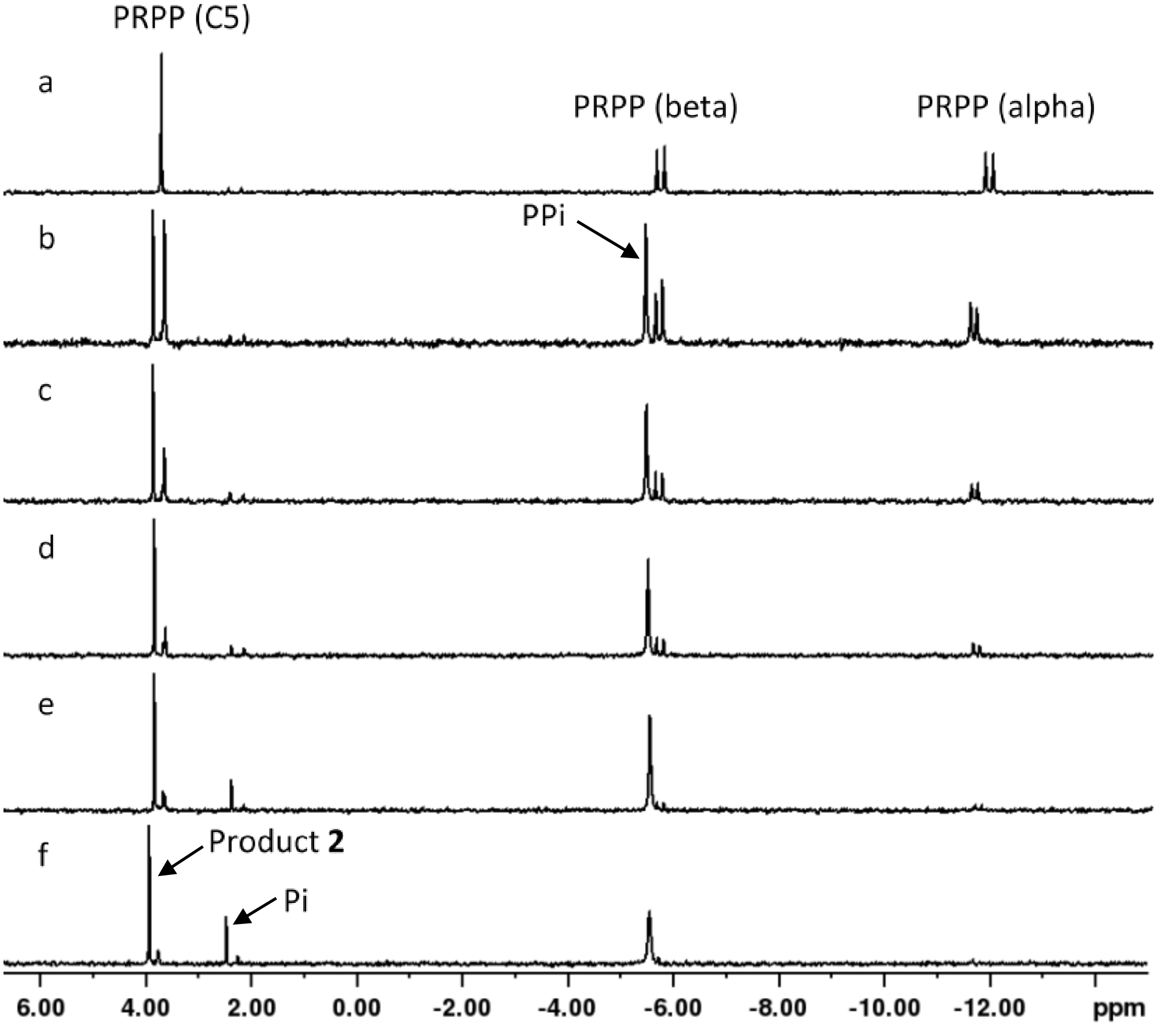

Figure S2. ^{31}P NMR spectra for the time course of the reaction containing 4.5 µM Cj1432$_{NMC}$, 5.0 mM compound **1**, 5.0 mM PRPP, and 5.0 mM $MgCl_2$ in 50 mM HEPEPS buffer, pH 8.0 at 30 °C. (a) Control containing only PRPP. (b) 12 min; (c) 19 min; (d) 25 min; (e) 48 min, and (f) 68 min.

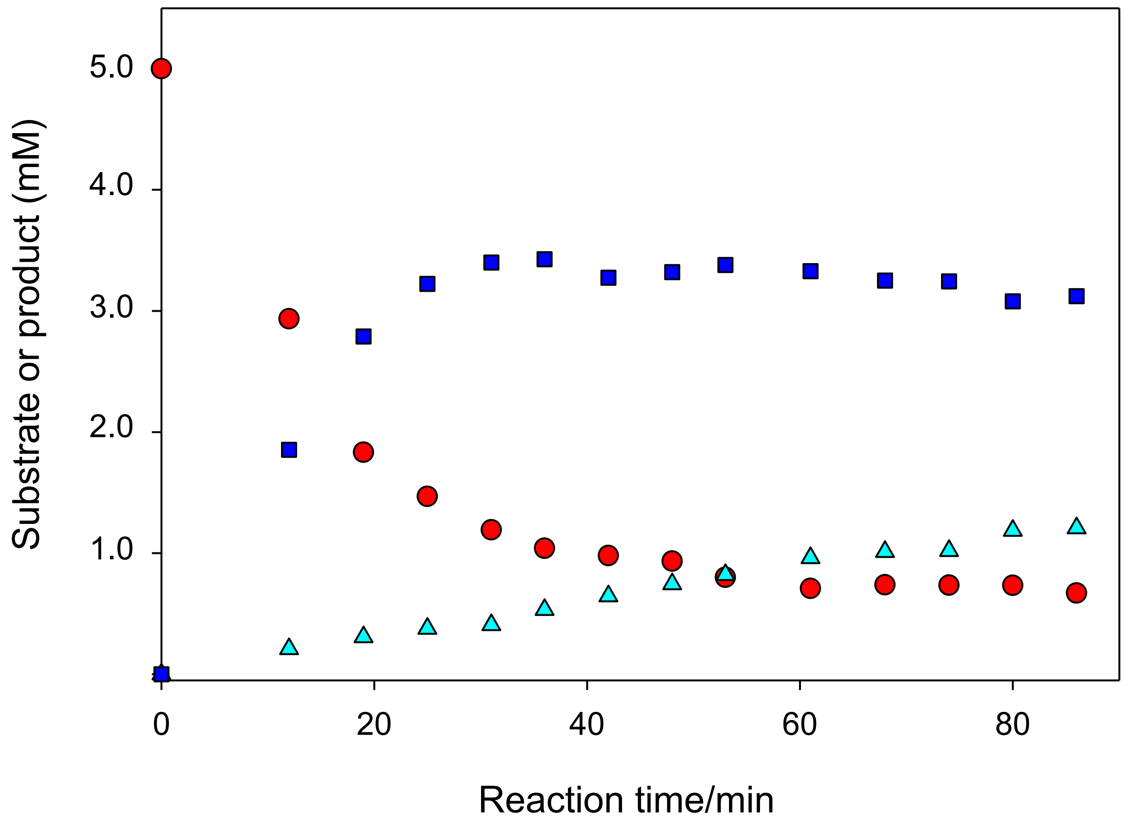

Figure S3: Time course for the formation of product **2** (blue square), formation of P_i (cyan triangle), and consumption of PRPP (red circle) from a reaction containing 5.0 mM compound **1** and 5.0 mM PRPP in the presence of 4.5 μM $Cj1432_{NMC}$ and 5.0 mM $MgCl_2$ in 50 mM HEPES buffer, pH 8.0 at 30 °C. The concentrations of P_i, PRPP, and product **2** were determined from changes in the ^{31}P NMR spectra taken as a function of time (see **Figure S2**).

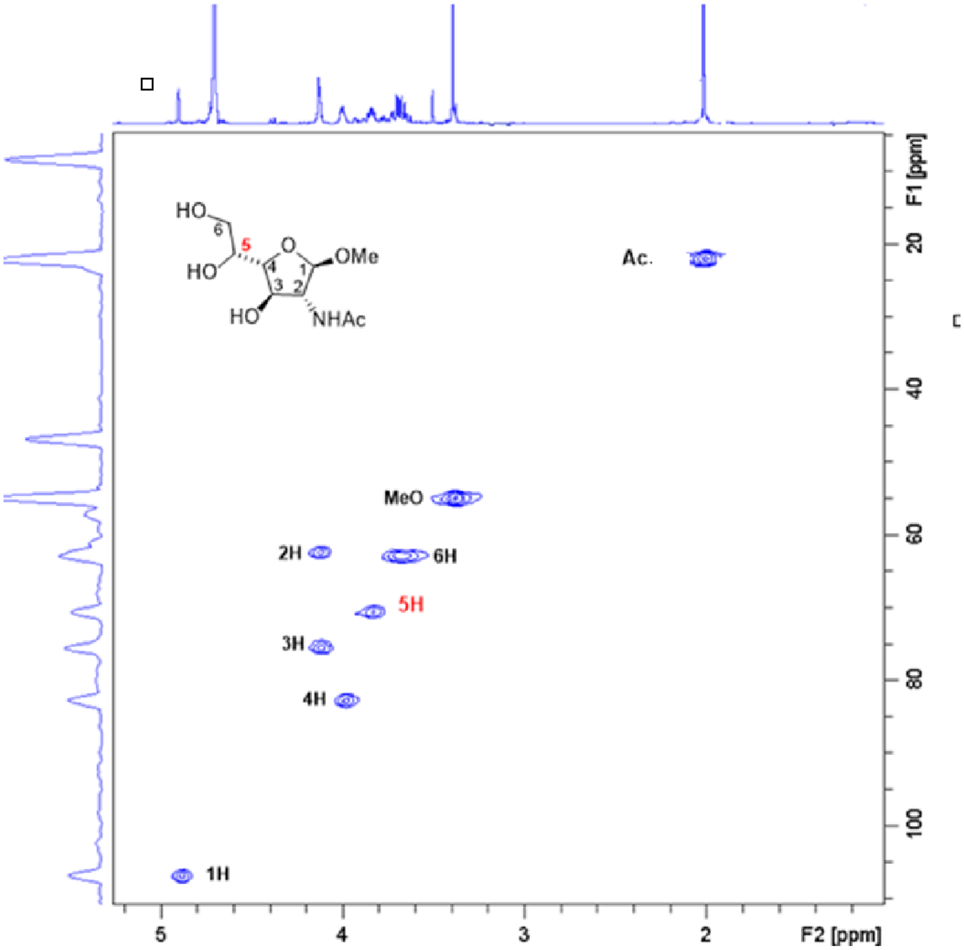

Figure S4: HSQC spectrum of methyl 2-acetamido-2-deoxy-β-D-galactofuranoside **1** in D_2O.

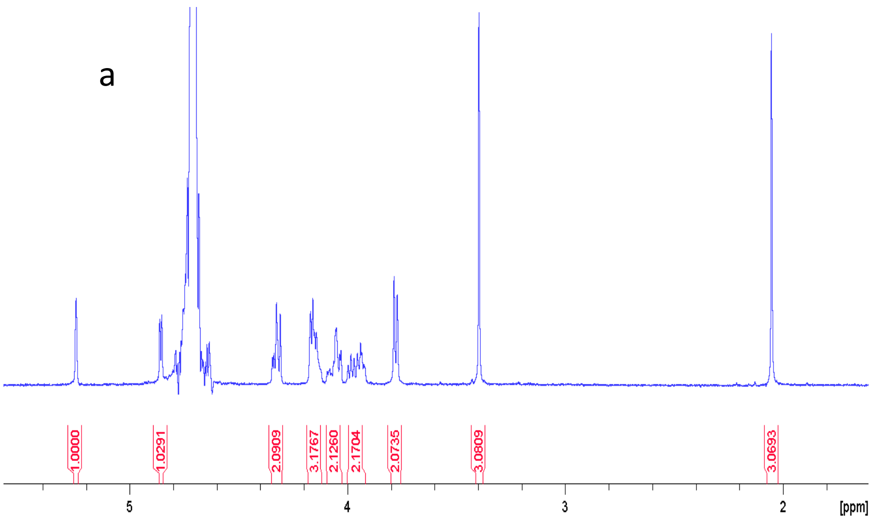

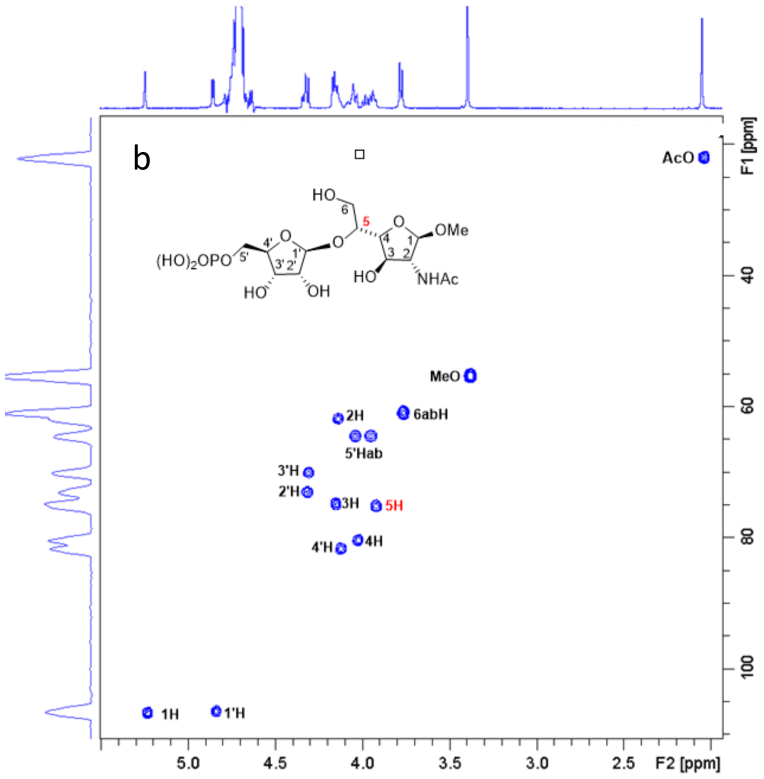

Figure S5: ^{1}H NMR spectrum (panel **a**) and HSQC spectrum (panel **b**). for disaccharide **2** in D_2O.

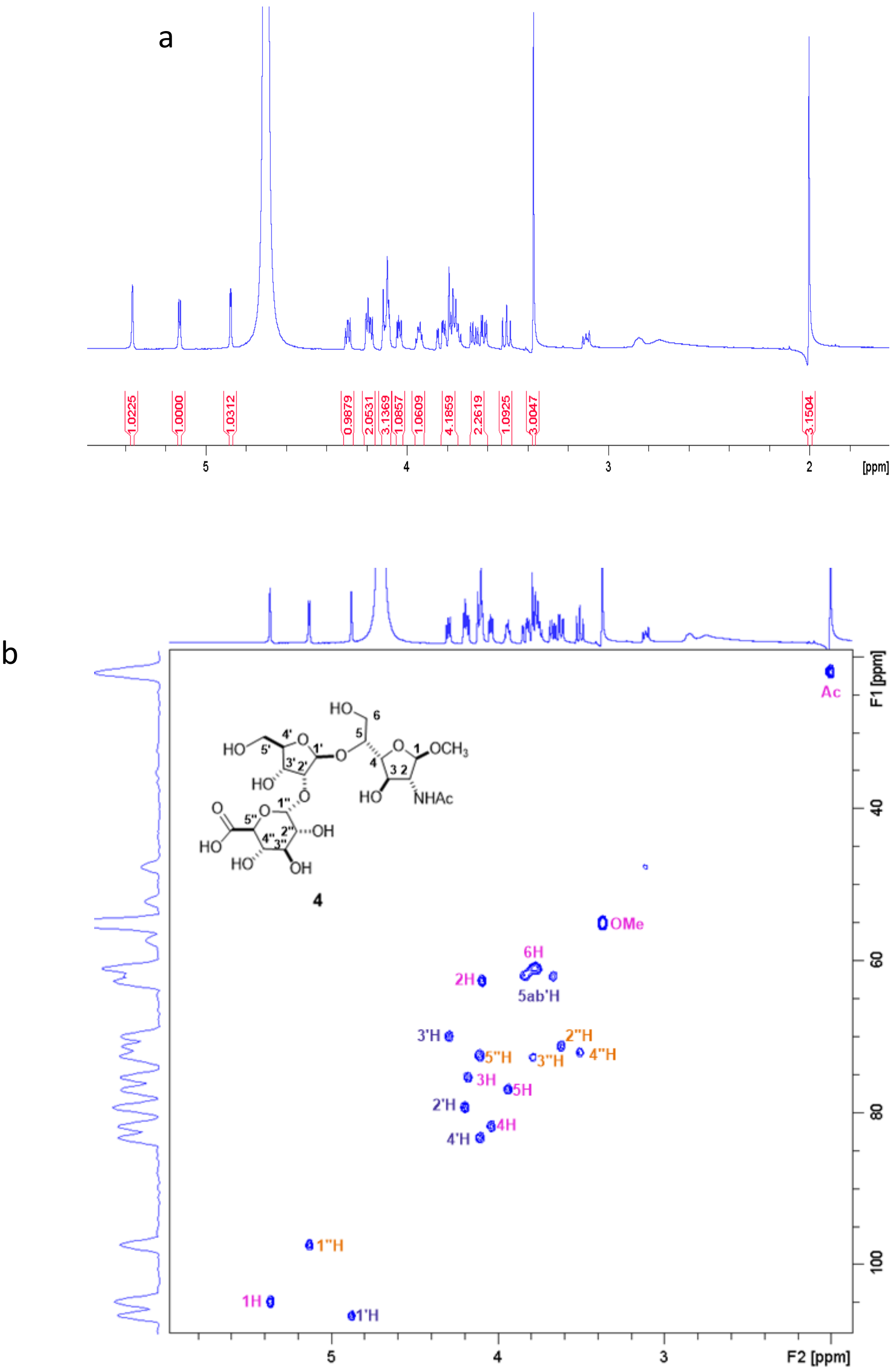

Figure S6. ^{1}H spectrum (panel **a**) and ^{1}H-^{13}C HSQC spectrum (panel **b**) of trisaccharide **4** in D_2O.

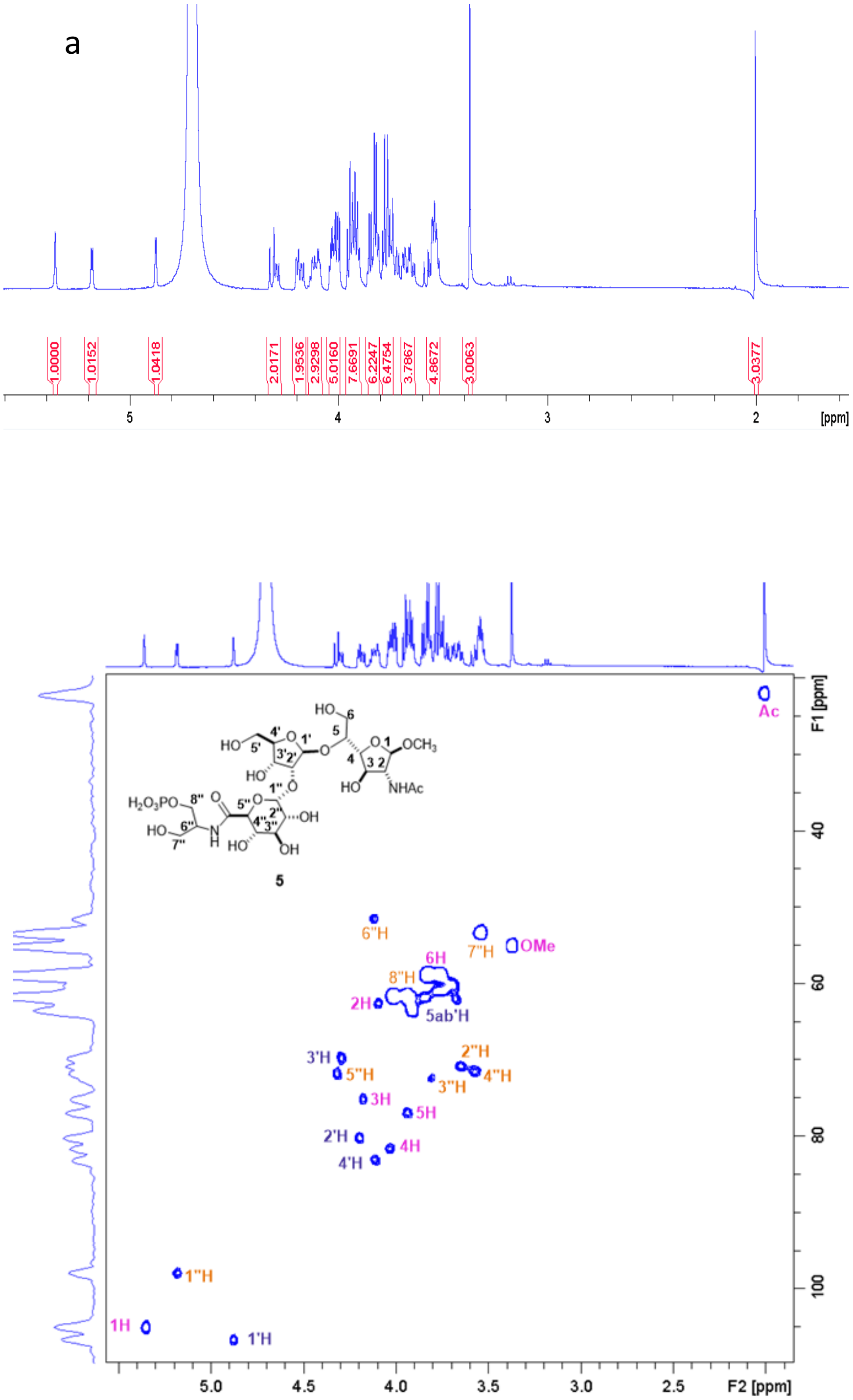

Figure S7. ^1H spectrum (panel **a**) and ^1H-^{13}C HSQC spectrum (panel **b**) of trisaccharide **5** in D_2O.

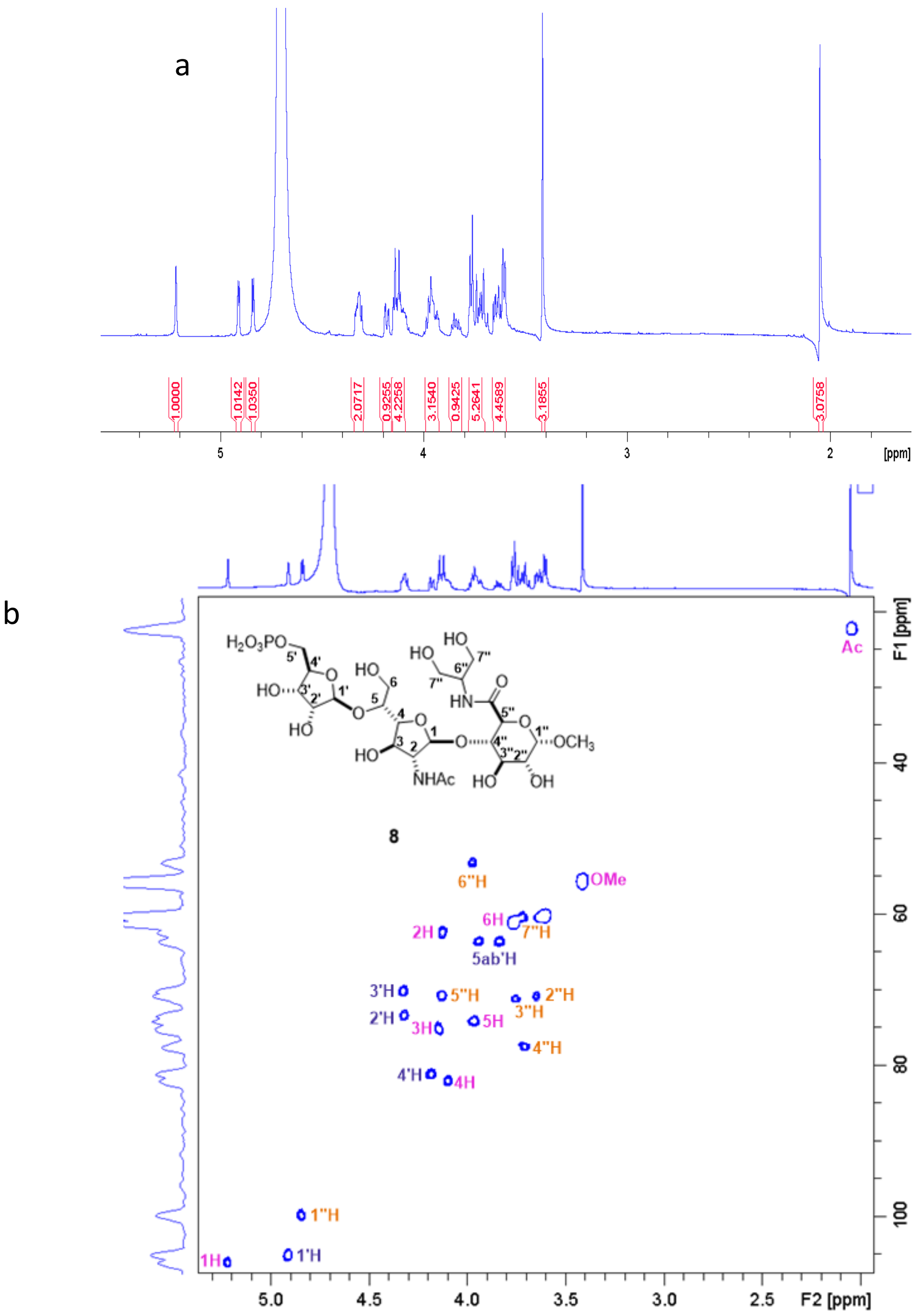

Figure S8. ^1H spectrum (panel **a**) and ^1H-^{13}C HSQC spectrum (panel **b**) of trisaccharide **8** in D_2O.

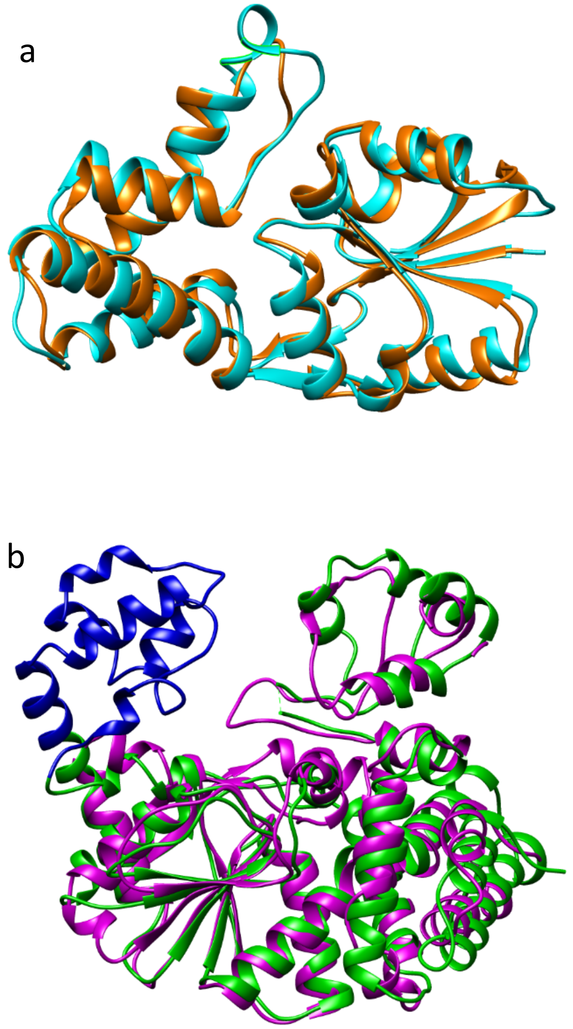

Figure S9: Comparison of the predicted AlphaFold2 structure of Cj1432 (AF-Q0P8I2-F1-v4) and the capsule polymerase Bcs3 (PDB id: 8A0C). (a) Superposition of the middle domain of Cj1432 (orange, residues 369-572) with the CrpP domain of Bcs3 (PDB id: 8A0C; cyan, residues 381-591). The calculated root mean square deviation (RMSD) for 183 pruned Cα pairs is 1.10 Å and 2.05 Å across all pairs. The sequence identity of the central domains of Cj1432 and 8A0C is 31%. (b) Superposition of the C-terminal domain of Cj1432 (purple, residues 574-907) and the CriT domain of Bcs3 (PDB id: 8A0C; green, residues 596-1014). The calculated root mean square deviation (RMSD) for 155 pruned Cα pairs is 1.18 Å and 4.23 Å across all pairs. The sequence identity of the C-terminal domains of Cj1432 and CriT is 25%. Compared to the C-terminal domain of Cj1432, the CriT domain has an extra loop (blue, residues 720-791) which is inserted between residues 687 and 688 of Cj1432.

```
                   D374
Cj1432      -LFSFDIFDTLISRNIAKPSAVFLIMKQK--MRNMDFPLNLVKNFDRIRVEVEQYYYRNV   426
crpP        ELHTFDIFDTLIRRSTLRPFSIFDYVRDKAKASGIKFPLALTENWINVRNRAEHDVRDIM   440
             *.:******** *.  :* ::*  :::*    .:.*** *.:*: .:* ..*:     :
                   D386

Cj1432      CKNK------YEDTNFDEIYNLLQQNFSLSFQQKEELMKLEINTEKETLYPIKKNIELVE   480)
CrpP        RKTTFERQSDKIEITLDDIYTRLQKNLLLTDEQTDFLKQAEIEAEIAHVEPIQKRINYLF   500)
             *..        : .:*:**. **:*: *: :*.: * : **::*   : **:*.*: :


Cj1432      ELIKNEKRVVLISDMYFSSSIIRTFLNKFSPIFNNIPIYMSSEFRLKKNSGNLFKAILNL   540
CrpP        SLKAKGHDVAMASDMYLPEDVIYKMLDRADTRLREIPLYLSSTIGYQKSTGKLYQHIFFD   560
             *  : : *.: ****: ..:* .:*:: .  :.:**:*:** :  :*.:*:*:: *:


Cj1432      EKVDPKKWIHCGDNWVGDYLKPSNLEISTNFYIN    574
CrpP        LDYQYSRWTHYGDNKHADGSVPRRLGIQTAV---    591
             . : ..:* * ***  .*   * .* *.* .
```

Figure S10a. Sequence alignment of the central domain of Cj1432 (Cj1432$_M$: residues 368-574) and the CrpP domain of Cps3D (residues: 381-591) (31%). Residue D386 in CrpP is a key residue for the activity and conserved in Cj1432$_M$ as D374. The sequence alignment was performed using Clustal Omega.

```
                                      
Cj1432        NQLLPYEEFALNRN-----------SLDMDLQKIIGISKKIRLENTLTNLQEIGVSFGAP 622
CriT          -DFIPFENAMVNAMDNYNRYPAYQLATKMHRY-RTQLVQENGFGNTLFETKYYNYAYVGA 653
               :::*:*:  :*             :  .*.      : ::  : *** : :  . :: .

                                      D649
Cj1432        MLLPYVQWILNIALKNSIRCLYFIARDGYVLQKMTDMLIQAKKINIKTKYLYGSRESWRE 682
CriT          SFVPYINWAIKDAIKRGYETIYFISRDGHFLKQIADKIIEIRGYNVKTKYIYGSRKAWRL 713
               ::**::* :: *:*.. . :***:***:.*::::* :*: :  *:****:****::**
                                      D680

Cj1432        PFRNKDKL---------------------------------------------------- 690
CriT          PSFITKVDDETFWQFGNFVGMDSFEDLVKASYLSESELLSLFPEFESLRHAKHLRGEIAE 773
              *   ..
                                                         E713
Cj1432        ----------------------KIQLIDEYLDQEIDKQEIFAFVECCGTGETLDYIVKRI 728
CriT          NIRKIFKNSPAYHEKVLAIAAEKRKMVRQYIQQEINPKEKFAFVEFWGRGYTQDTFGRLL 833
                                    * ::: :*::***: :* *****  * * * * : : :
                                                         E818

Cj1432        ESNQQFKNMFFGSLYLYRSKL---NKTKTQSLFMLPLNENYTYGIELFVRSLQGQVLGYD 785
CriT          ND--AFGKEVKNPFYYVRSFTDDMGTSVRHNFILAP--QNFSFFEPIFAQTPYDSIPDYY 889
              :.   * : . . :*  **     ..:  :.::: *  :*:::   :*.::  ..: .*

Cj1432        KKDGRVIPVFDFLEGEALQKFRYDEYIDGVMLFMEY-IVKTDNYEKIFDNMNVTILY--- 841
CriT          EEKGRIEPIINHRDRSVS-----DLISEGLLKFTEDYLALNTQDEDYFDAALSQFNYQYQ 944
              ::.**: *:::. : ..      *   :*:: * *  :. . : *. **     : *

Cj1432        LNYLSNNYIDKKFIEIMGNVPFILNGVKDRVGIFAPRLNNK--------ITLDQKNSFFN 893
CriT          LNTPNDQFICNVFSELKDNISSF--GVE---KPYAPALTLKQLESITSKQELDKLTQSIP 999
              **  .:::* : * *: .*:  :  **:     :** *. *          **: .. :

Cj1432        WSVLRSCKDIR-VKY--   907
CriT          ISLS--KSDVKVIDYYN   1014
               *:    .*:: :.*
```

Figure S10b. Sequence alignment of the C-terminal domain of Cj1432 (Cj1432$_C$: residues 574-907) and the CriT domain of Cps3D (CriT residues: 596-1014). The conserved catalytic residues, D686 and E818, in CriT (highlighted in red) are conserved in Cj1432$_C$ as D649 and E713 (highlighted in red), respectively. The sequence alignment was performed using Clustal Omega.

References

1. Xiang, D. F.; Riegert, A. S.; Narindoshvili, T.; Raushel, F. M. Identification of the Polymerizing Glycosyltransferase Required for the Addition of D-Glucuronic Acid to the Capsular Polysaccharide of *Campylobacter jejuni. Biochemistry* **2024**, *64*, 581-590.
2. Xiang, D. F.; Narindoshvili, T.; Raushel, F. M. Functional Characterization of Two Polymerizing Glycosyltransferases for the Addition of *N*-acetyl-D-Galactosamine to the Capsular Polysaccharide of *Campylobacter jejuni. Biochemistry* **2024**, *64*, 591-599.
3. Riegert, A. S.; Narindoshvili, T.; Platzer, N. E.; Raushel, F. M. Functional Characterization of a HAD Phosphatase Involved in Capsular Polysaccharide Biosynthesis in *Campylobacter jejuni. Biochemistry* **2022**, *61*, 2431-2440.
4. Yan, R-B., Yang, F., Wu, Y., Zhang, L-H., Ye, X-Sh. An Efficient and Improved Procedure for Preparation of Triflyl Azide and Application in Catalytic Diazotransfer Reaction. *Tetrahedron Lett.* **2005**, *46*, 8993-8995.
5. Snitynsky, R. B.; Lowary, T. L. Synthesis of Nitrogen-Containing Furanose Sugar Nucleotide for Use as Enzymatic Probes. *Org. Lett.* **2014**, *16*, 212-215.
